# Rhizosphere Organic Anions Play a Minor Role in Improving Crop Species' Ability to Take Up Residual Phosphorus (P) in Agricultural Soils Low in P Availability

**DOI:** 10.3389/fpls.2016.01664

**Published:** 2016-11-07

**Authors:** Yanliang Wang, Tore Krogstad, Jihong L. Clarke, Moritz Hallama, Anne F. Øgaard, Susanne Eich-Greatorex, Ellen Kandeler, Nicholas Clarke

**Affiliations:** ^1^Division of Biotechnology and Plant Health, Norwegian Institute of Bioeconomy ResearchÅs, Norway; ^2^Department of Environmental Sciences, Norwegian University of Life SciencesÅs, Norway; ^3^Soil Biology Department, Institute of Soil Science and Land Evaluation, University of HohenheimStuttgart, Germany; ^4^Division of Environment and Natural Resources, Norwegian Institute of Bioeconomy ResearchÅs, Norway

**Keywords:** phosphorus, rhizosphere organic anions, rhizosphere APase, rhizosphere pH, rhizosphere water-soluble P

## Abstract

Many arable lands have accumulated large reserves of residual phosphorus (P) and a relatively large proportion of soil P is less available for uptake by plants. Root released organic anions are widely documented as a key physiological strategy to enhance P availability, while limited information has been generated on the contribution of rhizosphere organic anions to P utilization by crops grown in agricultural soils that are low in available P and high in extractable Ca, Al, and Fe. We studied the role of rhizosphere organic anions in P uptake from residual P in four common crops *Triticum aestivum, Avena sativa, Solanum tuberosum*, and *Brassica napus* in low- and high-P availability agricultural soils from long-term fertilization field trials in a mini-rhizotron experiment with four replications. Malate was generally the dominant organic anion. More rhizosphere citrate was detected in low P soils than in high P soil. *B. napus* showed 74–103% increase of malate in low P loam, compared with clay loam. *A. sativa* had the greatest rhizosphere citrate concentration in all soils (5.3–15.2 μmol g^−1^ root DW). *A. sativa* also showed the highest level of root colonization by arbuscular mycorrhizal fungi (AMF; 36 and 40%), the greatest root mass ratio (0.51 and 0.66) in the low-P clay loam and loam respectively, and the greatest total P uptake (5.92 mg P/mini-rhizotron) in the low-P loam. *B. napus* had 15–44% more rhizosphere acid phosphatase (APase) activity, ~0.1–0.4 units lower rhizosphere pH than other species, the greatest increase in rhizosphere water-soluble P in the low-P soils, and the greatest total P uptake in the low-P clay loam. Shoot P content was mainly explained by rhizosphere APase activity, water-soluble P and pH within low P soils across species. Within species, P uptake was mainly linked to rhizosphere water soluble P, APase, and pH in low P soils. The effects of rhizosphere organic anions varied among species and they appeared to play minor roles in improving P availability and uptake.

## Introduction

In order to produce enough food to feed the increasing global population, large amounts of mineral phosphorus (P) fertilizers have been used. Soluble P applied to agricultural soils as P fertilizers is readily sorbed to aluminum (Al) and iron (Fe) (hydr)oxides (in acid soils) or calcium (Ca) (in calcareous soils) exposed at the surfaces of soil constituents, leading to up to 80% of soil P being unavailable for uptake by most plants (Raghothama, 1999; Vance et al., 2003). For example, in Norwegian agricultural soils, although the average P surplus has been reduced by nearly 50% since 1985, the surplus is still about 15 kg P ha^−1^ year^−1^ (Eurostat, http://ec.europa.eu/eurostat/web/agri-environmental-indicators/farm-management-practices). Therefore, many arable lands have accumulated large reserves of residual P: In Norway, a relatively large proportion of cultivated soils is classified as high to very high in extractable P (Singh and Subramaniam, 1996). Only 3–25% of total P was extractable in ammonium lactate (P_AL_), which is an estimate of the plant-available P, whereas 34–68% of P was sorbed to Al, Fe, and Ca. In addition, organic P accounted for 17–51% of total P in different Norwegian soils (Singh et al., 2005). Improving the utilization of residual P accumulated in agricultural soils would help to reduce P fertilizer application and environmental stress. Hence, studies on how to mobilize the less-available residual P and improve P-acquisition efficiency are needed.

Higher plants have evolved root morphological and physiological strategies to improve P availability and enhance P uptake (Lambers et al., 2006; Faucon et al., 2015). Root morphological strategies include altered root mass distribution, root length, root surface area, and number and length of root hairs and lateral roots (Lambers et al., 2006, 2008; Lynch, 2011; Pedas et al., 2011). Root physiological traits that play active roles in enhancing P availability and uptake include the release of protons to change rhizosphere pH, which in turn changes soil P adsorption and desorption; the exudation of organic anions such as citrate and malate, which can mobilize both inorganic and organic P (Lambers et al., 2015); and the release of phosphatase enzymes, which hydrolyse soil organic P to release inorganic P (Tarafdar et al., 2001; George et al., 2004). Moreover, P uptake can also be improved by root-colonizing arbuscular mycorrhizal fungi (AMF), which increase the volume of the soil that can be explored by plant roots (Smith and Read, 2010). For some plant and AMF species, P uptake by mycorrhizal hyphae has been shown to be the dominant pathway for P acquisition when plant roots are colonized by AMF (Smith et al., 2003; Watts-Williams et al., 2015). Root released organic anions may play a key role in mobilizing less-available P (Ryan et al., 2001; Lambers et al., 2006), as shown by root released organic anions increasing under P deficiency (Hoffland et al., 1989; Gahoonia et al., 2000) and over-expression of organic anion synthase genes and organic anion transporters in several plants leading to enhanced P uptake ability (Lü et al., 2012; Wang et al., 2013). On the other hand, higher root organic anion exudation does not necessarily result in higher grain yield (Pandey et al., 2014). Moreover, root exudates do not relate consistently to P uptake from less available P sources (Pearse et al., 2007). Field experiments using near isogenic lines of wheat that differed in citrate efflux indicated that citrate efflux provided no consistent advantage for biomass production or yield (Ryan et al., 2014). The above reports suggest that further study is necessary to elucidate the role of root exudates in mobilizing plant less-available P.

In our previous study, we conducted a hydroponic experiment and found contrasting responses of root morphology and root-exuded organic acids to low P availability in three important food crops, *Brassica napus, Hordeum vulgare*, and *Solanum tuberosum*, which have divergent root traits (Wang et al., 2015). In the current study, we carried out an experiment using a mini-rhizotron culture system as described by James et al. (1985) and selected two dicot and two monocot widely cultivated crops (*Triticum aestivum, Avena sativa, Solanum tuberosum* and *Brassica napus*) to investigate the contribution of root-exuded organic anions to improving P uptake in agricultural soils low in P availability.

For these four crops grown in three soils obtained from long-term fertilization field plots in Norway, we addressed three hypotheses: (1) Low P availability will stimulate plant roots to release more organic anions and APase to rhizosphere soil; (2) the amounts of rhizosphere organic anions and APase will have positive correlations with rhizosphere plant-available P fractions and P uptake by plants in low P soils; (3) different crops will show differences in root released organic anions and APase in terms of using residual P from agricultural soils. The ultimate goal of this study is to increase understanding of the contribution of root-released organic anions and APases to P uptake in low P availability agricultural soils in common crops.

## Materials and methods

### Soil sampling, preparation, and analysis

The soils for the rhizotron experiment were collected from the plow layer (0–20 cm) of a clay loam and a loam of two long-term fertilization trials in southeastern Norway (Kristoffersen and Riley, 2005). The clay loam (26% clay, 38% silt, 36% sand) was collected from field plots at Ås, Akershus county (59°39′ N, 10°45′ E), which had received either 48 (P high, soil A_HP_) or 0 (P low, soil A_LP_) kg P ha^−1^ year^−1^ as single superphosphate since 1966. Both P treatments received 100 kg N ha^−1^ year^−1^ as calcium nitrate and 100 kg K ha^−1^ year^−1^ as potassium chloride. The loam (soil B:14% clay, 34% silt, 52% sand) was collected from a field in Møystad, Hedmark county (60°47′ N, 11°10′ E), which had only received nitrogen (N) and potassium (K) fertilizers since 1922 (100 kg N ha^−1^ year^−1^ as calcium nitrate and 120 kg K ha^−1^ year^−1^ as potassium chloride).

The soils were air-dried at 30°C, passed through a 3.15 mm sieve, homogenized, and subsequently analyzed in order to make an initial characterization of the soils (Table 1). The pH was measured in water extract, solid: solution ratio of 1:2.5 (v/v), plant-available P (ammonium lactate extractable P, i.e., P_AL_) was determined according to Egnér et al. (1960) and easily releasable P was extracted in 0.0025 M CaCl_2_, solid: solution ratio of 1:20 (w/v), hereafter called water-soluble P, WSP). According to the guidelines used in Norway (Krogstad et al., 2008), P_AL_-values below 30 mg kg^−1^ are considered low, whilst those above 140 mg kg^−1^ are considered very high. This standard was used in the present study. The pH of soils A_HP_ and A_LP_ was adjusted from 5.4 and 5.0, respectively to ~6.5 by adding moderate amounts of CaCO_3_before use. No P fertilizers were applied to the soils but all other basic nutrients were provided as follows, in mg kg^−1^ soil: K, 70.2; N, 70; Mg, 9.6; S, 29.9; Zn, 0.65; Mo, 0.48; Cu, 0.32; Fe, 5.6; Mn, 1.1; B, 0.11; Na, 0.12. For N, K, Mg, and S, weighed K_2_SO_4_, NH_4_NO_3_, and MgCl_2_ powders were mixed thoroughly with the soils assuring a homogeneous distribution of nutrients in the soils. For micronutrients, chemicals were dissolved in water, and then applied to each pot to ensure that plant growth was not limited by these micronutrients.

**Table 1:** **Properties of the selected soils**.

**Soil**	**A_HP_**	**A_LP_**	**B**
pH (H_2_O)	5.4	5.0	6.0
Total C (%)	2.89	2.25	2.93
Total N (%)	0.28	0.24	0.33
AL extractable P (mg kg^−1^ soil)	150	37	33
AL extractable Ca (mg kg^−1^ soil)	1700	1300	1900
Oxalate extractable P (mg kg^−1^ soil)	1000	520	480
Oxalate extractable Al (mg kg^−1^ soil)	2500	1900	1200
Oxalate extractable Fe (mg kg^−1^ soil)	6100	4900	4400
Water soluble P (mg kg^−1^ soil)	10.64	0.81	1.78
P sorption capacity (mmol kg^−1^ soil)	100.9	79.1	61.6
Degree of P saturation (%)	32.0	21.2	25.1

*AL, ammonium lactate. P sorption capacity and degree of P saturation were calculated according to Maguire et al. (2001)*.

### Plant materials, growth, and experimental design

Canola (*B. napus* cv. MARIE), wheat (*T. aestivum* cv. AINO), oat (*A. sativa* cv. BELINDA), and micropropagated seedlings of potato (*S. tuberosum* cv. PIMPERNEL) were grown in 20 cm × 20 cm × 1 cm mini-rhizotrons consisting of Plexiglas plates (James et al., 1985). The experiment was conducted in a greenhouse with 18°C/15°C day/night temperature with a 16 h photoperiod at a light intensity of 200 ± 20 μmol m^−2^ s^−1^ and 50–75% relative humidity. After filling each mini-rhizotron with ~0.5 kg fertilized homogenized soils, water was added to achieve a soil moisture level of 25% (w/w). Mini-rhizotrons were wrapped in black plastic bags to avoid light exposure. Five surface-sterilized seeds were sown in each mini-rhizotron and three uniform seedlings were kept in each mini-rhizotron after germination. For potato, three tissue culture-derived seedlings of around 10 cm height were used. There were four replicates and one mini-rhizotron without plant for each soil was set as control-unplanted bulk soil. Deionized water (15–40 mL) was given daily to keep the soil surface moist. Through the whole experimental period, the plants were irrigated according to weight loss, and the position of the mini-rhizotrons was changed randomly every week.

### Plant harvest and rhizosphere soil sampling

Three plants in each mini-rhizotron were harvested 5 weeks after germination and no potato root tuber was produced during the 5-week experiment. At harvest, the intact plants were carefully removed from the mini-rhizotrons and divided into shoots and roots. The roots were first shaken slightly to remove excess soil. The soil remaining attached to the roots was defined as rhizosphere soil (e.g., Veneklaas et al., 2003). For each mini-rhizotron, about 30 g rhizosphere fresh soil was carefully sampled using tweezers and spoons. The rhizosphere soil was divided into two groups, one air-dried for analysis of rhizosphere soil pH, P_AL_ and water-soluble P, and another stored at −20°C for microbial P immobilization and soil enzyme activity measurements.

### Rhizosphere organic anion collection and determination

After subsamples of rhizosphere soil were obtained, the entire root system with the remaining rhizosphere soil was transferred into a container with a known volume of 0.2 mM CaCl_2_ solution to ensure cell integrity. Roots were then gently and carefully dunked for 60–90 s to get rhizosphere extract (Pearse et al., 2007; Pang et al., 2015). A subsample of the rhizosphere extract was taken and filtered through a Phenex regenerated cellulose syringe filter (pore size 0.45 μm, filter diameter 15 mm) (Phenomenex, Torrance, CA, USA). Micropur (0.01 g L^−1^, Katadyn Products, Kemptthal, Switzerland) was then added to the solution to inhibit the activity of microorganisms (Cheng et al., 2014). The collected rhizosphere extracts were immediately frozen and stored at −20°C until analysis with liquid chromatography triple quadrupole mass spectrometry (LC–MS/MS, Waters, Milford, MA, USA and Micromass, Manchester, UK) was carried out. The root systems were then washed thoroughly to remove remaining soil and sub-sampled for AMF detection. The remaining extract containing the rhizosphere soil in the container was centrifuged at 4000 rpm and the supernatant transferred to a new container. The container with soil was then placed in a 65°C oven for 2 weeks. The rhizosphere soil inside the container was then weighed.

Before analysis with LC-MS/MS, 910 μL extract was taken out of each sample to a separate vial, 50 μL deuterium-labeled succinic acid (0.2 μg) were added to be used as an internal standard (IS), and each vial was acidified with 40 μL concentrated formic acid. The LC-MS/MS analysis was performed as described previously (Wang et al., 2015). The concentrations were determined by comparison with their standard concentration measurements and further calculated based on the root dry weight or rhizosphere soil dry weight.

### Root colonizing mycorrhizal fungi

After sampling of rhizosphere extract, fresh root subsamples were randomly taken and examined for AMF and other root inhabiting fungi (non-AMF). Roots were maintained in 10% (w/v) KOH for 3 days at ~25°C, and then stained in a 0.1% aniline blue solution for 1 h and distained/stored in lactoglycerol (Vierheilig et al., 1998). The line intersect method was used to assess the percentage of root length colonized by AMF and non-AMF (Giovanetti and Mosse, 1980). For each root sample, ten 1-cm pieces were randomly selected and five fields of vision were examined in each 1-cm root section at 100x microscopy; thus, 50 fields of vision were examined for each sample. The colonization percentage was calculated as the ratio of the colonized sections to the total sections examined. Identification of AMF was based on observations of arbuscules, and roots where only intraradical or extraradical hyphae or vesicles were observed were defined as non-AMF.

### Plant phosphorus determination

Shoots and roots were dried at 65°C for 48 h, and dry weight (DW) was measured. Root mass ratio was calculated as the ratio of root DW to the total plant DW. Shoot and root P concentrations were determined by inductively coupled plasma atomic emission spectroscopy (AtomComp 1100, Thermo Jarrell-Ash, MA, USA) according to Ogner et al. (1999) after digestion in a mixture of 65% (v/v) HNO_3_/72% (v/v) HClO_4_ (5: 1, v/v) at 220°C in a microwave oven. Shoot and root P contents were calculated by P concentrations × shoot or root DW.

### Rhizosphere soil enzyme activities

Acid phosphomonoesterase (EC 3.1.3.2), β-glucosidase (EC 3.2.1.21), β-xylosidase (EC 3.2.1.37), and N-acetyl-β-glucosaminidase (EC 3.2.1.52) activities were measured by multi-substrate microplate-scale fluorometric assays based on the use of 4-methylumbelliferone (MUF) as described by Giacometti et al. (2014). A subsample of 1 g moist soil from the rhizosphere was placed into a 100 mL beaker with 50 mL de-ionized water and the soil was then dispersed with an ultrasonic bar for 2 min. Fifty microliter of soil suspension, 50 μL MES buffer, and 100 μL substrate (4-methylumbelliferyl-phosphate, 4-methylumbelliferyl-β-D-glucoside, 4-methylumbelliferyl-β-D-xylopyranoside, and 4-methylumbelliferyl-N-acetyl-β-D-glucosaminide, all 1 mM) were pipetted into one well of a 96-well black polystyrene microplate. The microplates were covered and incubated in the dark at 30°C. Fluorescence intensity was measured using a microplate reader (Flx® 200, Biotek Instruments, Winooski, USA) with 360 nm excitation and 460 nm emission filters. Measurements were made immediately after the plate was set up and then every 30 min over a 3 h incubation period. Each sample had three analytical replicates and the concentrations were determined by comparison with their standard concentration measurements. Rates of fluorescence increase were converted to enzyme activity (nmol MUF gDM^−1^ h^−1^) according to German et al. (2011).

### Soil microbial biomass phosphorus (p_mic_)

Concentration of P held in the microbial biomass (P_mic_) was determined by the anion exchange membrane-based fumigation extraction method as described by Kouno et al. (1995) with slight modifications. Moist soil (equivalent to 2 g dry matter) samples were shaken (160 rpm) with two 1 cm × 2 cm resin membrane strips in (1) 30 mL distilled water, (2) 30 mL distilled water + 1 mL hexanol, and (3) 30 mL distilled water + 1 mL P solution with a known P concentration as a spike to correct for soil sorption of P released during fumigation-extraction. After shaking for 17 h at room temperature, P adsorbed on resin strips was extracted by transferring them into a clean tube, followed by adding 20 mL 0.5 M HCl and shaking for 1 h under the same conditions. Finally, P concentration in HCl solution was determined photometrically (Murphy and Riley, 1962).

### Statistical analyses

R software (version 3.2.3) was used for data analyses. Two-way ANOVAs were used to study main effects of soil, plant species, and their interaction on all parameters involved in this study, followed by pairwise Tukey's honest significant difference tests for multiple comparisons, along with the minimum significant difference (MSD) at *p* < 0.05 (presented in figure captions). Simple linear regressions were used to estimate the relationships among response variables. Within each soil type, linear regressions were made across species. Within species, linear regressions were made across either similar soil properties (A_HP_ and A_LP_) or similar P availability (A_LP_ and B), or for all three soils.

## Results

### Nitrogen: phosphorus ratio

**Figure 1:**
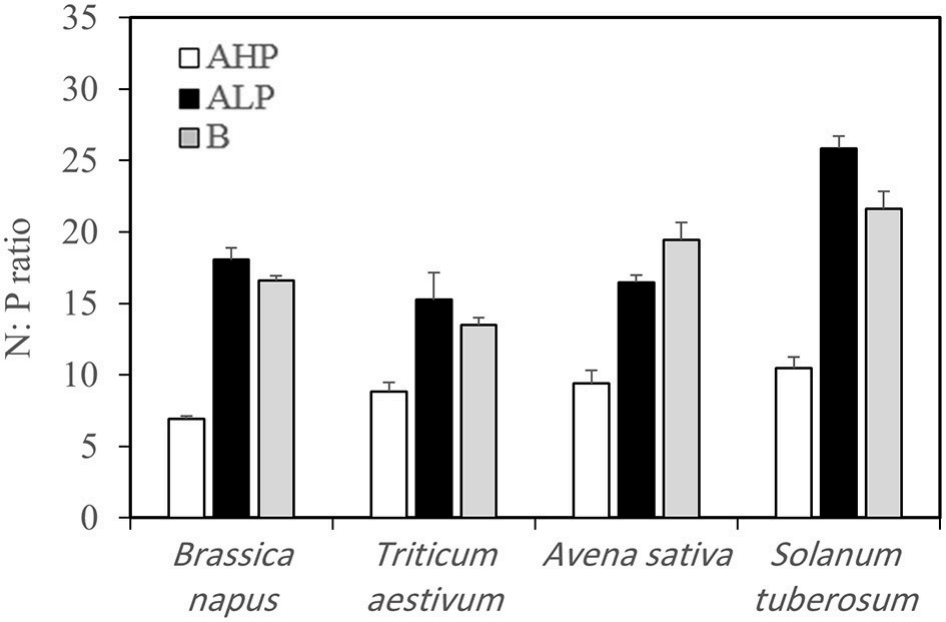
**N:P ratios of the aboveground tissues of ***Brassica napus***, ***Triticum aestivum***, ***Avena sativa***, and ***Solanum tuberosum*** grown in soils A_**HP**_ (white bars), A_**LP**_ (black bars), and B (gray bars)**. Error bars indicate SE (*n* = 4). There was a significant interaction between species and soils for N:P ratio (*p* < 0.001, MSD _0.05_ = 1.80).

No P was applied in our system. In order to confirm that P was the limiting factor for plant growth in low P soils, we calculated the N:P ratio in aboveground plant tissues (Table 2 and Figure 1). The N:P ratio varied from 6.9 to 10.5, 15.3 to 25.8, and 13.5 to 21.6 when soils A_HP_, A_LP_, and B were used as growth medium, respectively. According to van Duivenbooden et al. (1995), the N:P ratio of agricultural crops is in general between 6 and 8, and plants can be diagnosed as P limited when the N:P ratio is above 14. Hence, our soils A_LP_ and B were P deficient and soil A_HP_ was P sufficient.

**Table 2:** **Significance of a two-way ANOVA analysis for measured parameters**.

	**Species**	**Soils**	**Species × soils**
N:P ratio	^***^	^***^	^***^
Root colonization by AMF	^***^	ns	ns
Shoot dry weight	^***^	^***^	^***^
Total dry weight	^***^	^***^	^**^
Root mass ratio	^***^	^**^	^**^
Shoot P concentration	^***^	^***^	^**^
Root P concentration	^***^	^***^	^***^
Total P content	^***^	^***^	ns
Citrate	^***^	ns	ns
Malate	^***^	ns	^*^
Total organic anions	^***^	ns	ns
Rhizosphere pH	^***^	^***^	^***^
Rhizosphere plant available P	ns	^***^	^*^
Rhizosphere water soluble P	^***^	^***^	^**^
Soil microbial P	^**^	^***^	^**^
Rhizosphere acid phosphatase activity	^***^	^***^	^***^

Significant effects are indicated for plant species, soils and their interactions (ns, not significant;

* p < 0.05;

** p < 0.01;

*** *p < 0.001)*.

### Biomass response and partitioning

**Figure 2:**
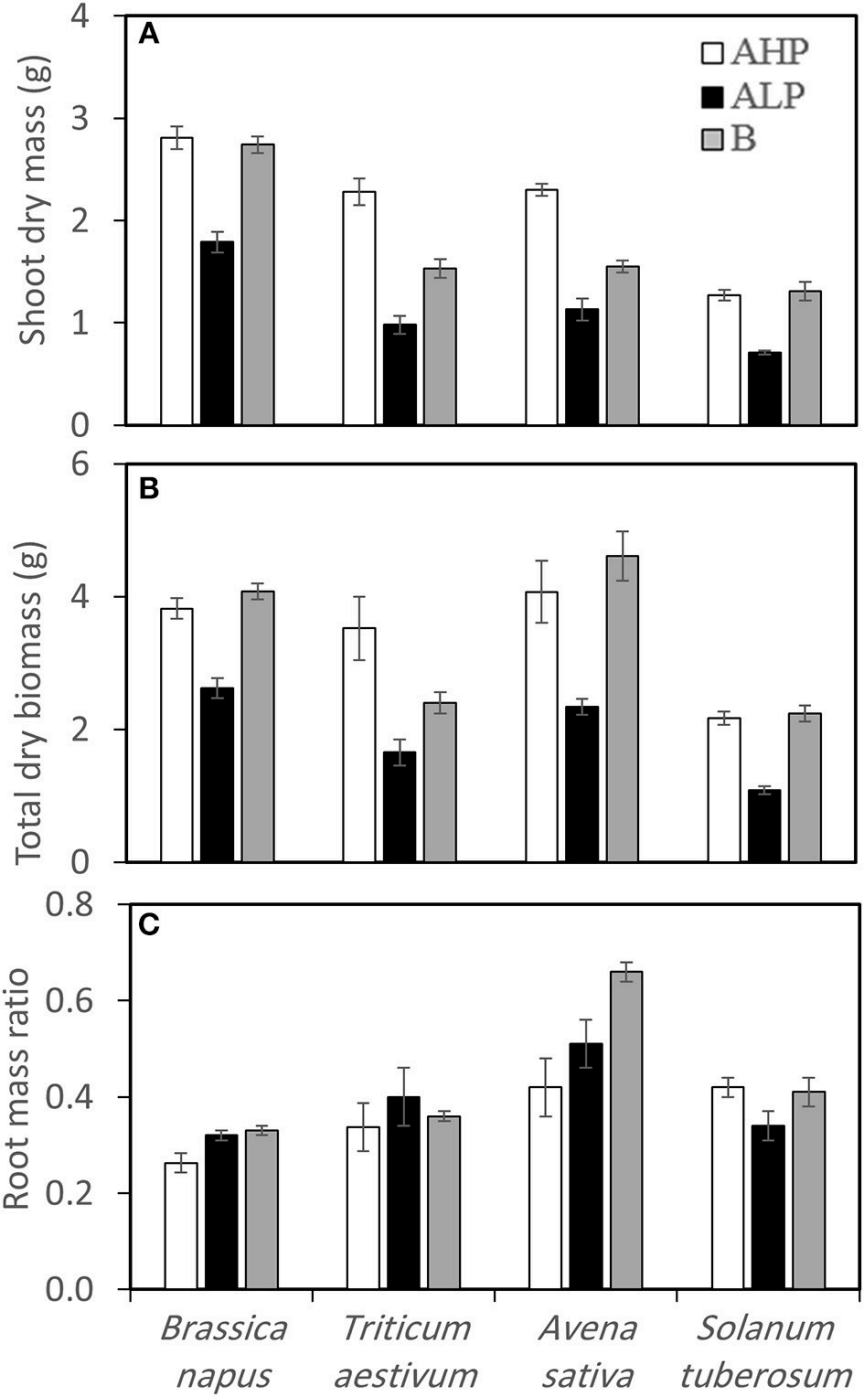
**(A)** Shoot dry weight, **(B)** total dry weight, and **(C)** root mass ratio (dry matter basis) of *Brassica napus, Triticum aestivum, Avena sativa*, and *Solanum tuberosum* grown in soils A_HP_ (white bars), A_LP_ (black bars), and B (gray bars). Error bars indicate SE (*n* = 4). There was a significant interaction between species and soils for shoot dry weight (*p* < 0.001, MSD _0.05_ = 0.17 g), total dry weight (*p* < 0.01, MSD _0.05_ = 0.48 g) and root mass ratio (*p* < 0.01, MSD _0.05_ = 0.07).

Shoot and total dry biomass varied significantly among soils and species (Table 2 and Figures 2A,B). All crops showed significant decrease of both shoot and total biomass in soil A_LP_, compared with soil A_HP_; however, crops grown in soil B showed almost the same total biomass as those grown in soil A_HP_, except *T. aestivum* (Figure 2). Dicots had similar shoot biomass in soil A_HP_ and soil B while monocots had lower shoot biomass in soil B, compared with soil A_HP_. *B. napus* accumulated the most shoot biomass in all soil types (average 2.81 g, 1.79 g, and 2.74 g in soils A_HP_, A_LP_, and B, respectively) while *S. tuberosum* accumulated the least shoot biomass (average 1.27 g, 0.71 g, and 1.31 g in soils A_HP_, A_LP_, and B, respectively); *T. aestivum* and *A. sativa* had similar amounts of shoot biomass in all soils.

Different root mass ratio (root DW: total DW) patterns were observed in the four crops (Figure 2C). Compared with soil A_HP_, *B. napus* showed a significant increase of root mass ratio in soil A_LP_ and soil B, by 23 and 27%, respectively while *A. sativa* showed 21 and 57% increase, respectively. *T. aestivum* did not show any significant differences in root mass ratios between soils and *S. tuberosum* showed a decrease of root mass ratio by 19% when plants were grown in soil A_LP_ compared with A_HP_, but showed about the same value when plants were grown in soil B and soil A_HP_.

### P response and P uptake by plants

**Figure 3:**
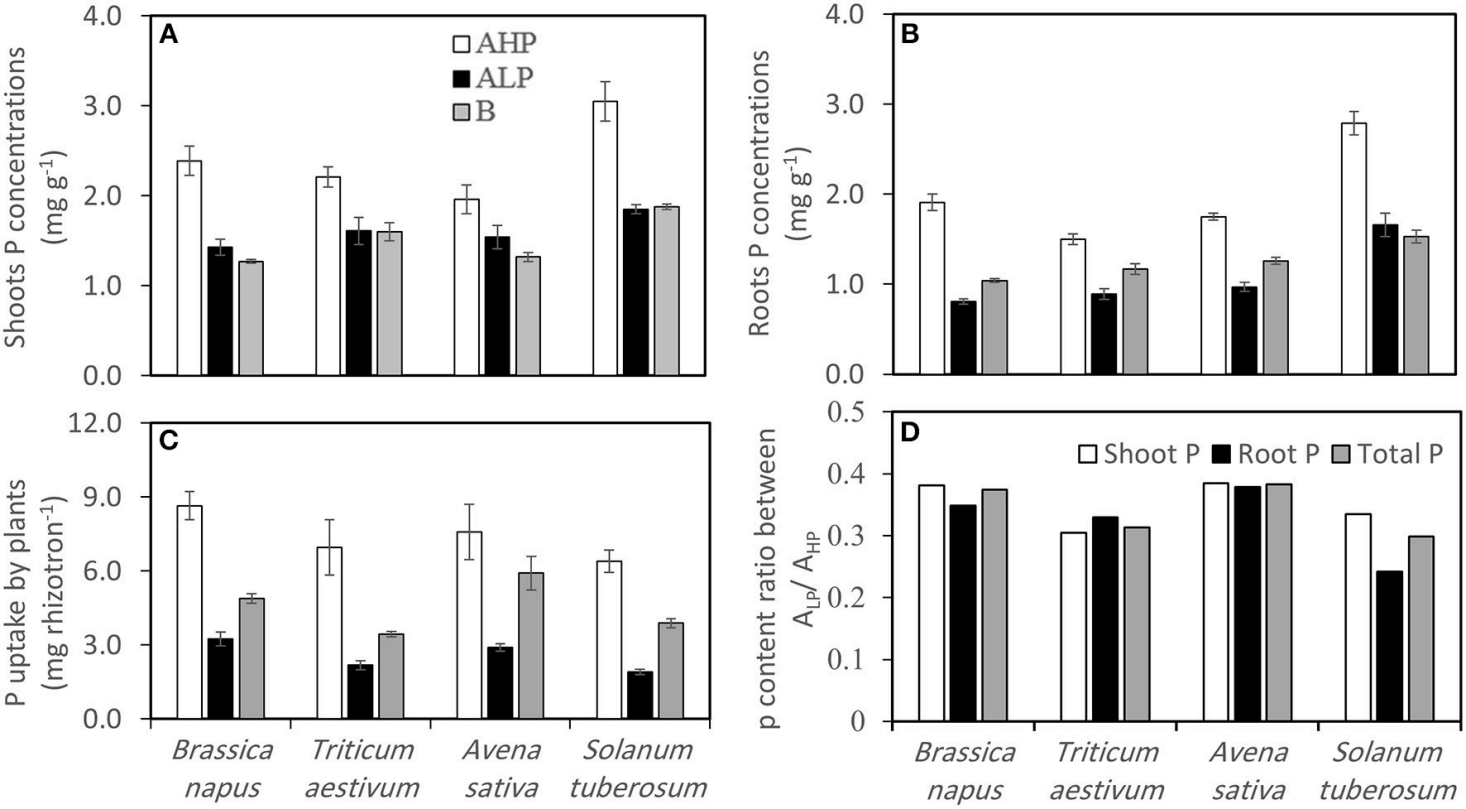
**(A)** Shoot P concentrations, **(B)** root P concentrations, **(C)** total P uptake of *Brassica napus, Triticum aestivum, Avena sativa*, and *Solanum tuberosum* grown in soils A_HP_ (white bars), A_LP_ (black bars) and B (gray bars), and **(D)** P content ratio between soils A_LP_/A_HP_ for shoot P (white bars), root P (black bars), and total P (gray bars). Error bars indicate SE (*n* = 4). There was a significant interaction between species and soils for shoot P concentrations (*p* < 0.01, MSD _0.05_ = 0.23 mg g^−1^), and root P concentrations (*p* < 0.001, MSD _0.05_ = 0.14 mg g^−1^).

Shoot and root P concentrations varied significantly, and were unsurprisingly highest when plants were grown in soil A_HP_ for all crops (Table 2 and Figures 3A,B). For all three soils, *S. tuberosum* had the highest shoot and root P concentrations, while *B. napus* had the lowest shoot and root P concentrations in soils A_LP_ and B. Generally, the shoot P concentrations in soil B were equal (*S. tuberosum* and *T. aestivum*) or a little bit lower (*B. napus* and *A. sativa*) than those in soil A_LP_, whereas the root P concentrations in soil B tended to be higher than in soil A_LP_ with the exception of *S. tuberosum*.

Shoot and root P content followed the order soil A_HP_ > B > A_LP_ for all species, except for root P content in *A. sativa* (Figure S1). The total P acquired by plants also varied significantly among soils and species (Table 2 and Figure 3C). Unsurprisingly, the greatest amount of P was removed from soil A_HP_, and the least was removed from soil A_LP_. For soil A_LP_, *B. napus* acquired the most P (3.24 mg/mini-rhizotron) while *S. tuberosum* obtained least P (1.91 mg/mini-rhizotron), whilst for soil B, *A. sativa* obtained the greatest P (5.92 mg/mini-rhizotron) and *T. aestivum* obtained the least (3.44 mg/mini-rhizotron). The P uptake ratio between A_LP_/A_HP_ indicated that *B. napus* and *A. sativa* performed better in acquiring P under low P conditions than the two other species (Figure 3D).

### Rhizosphere organic anion response

**Figure 4:**
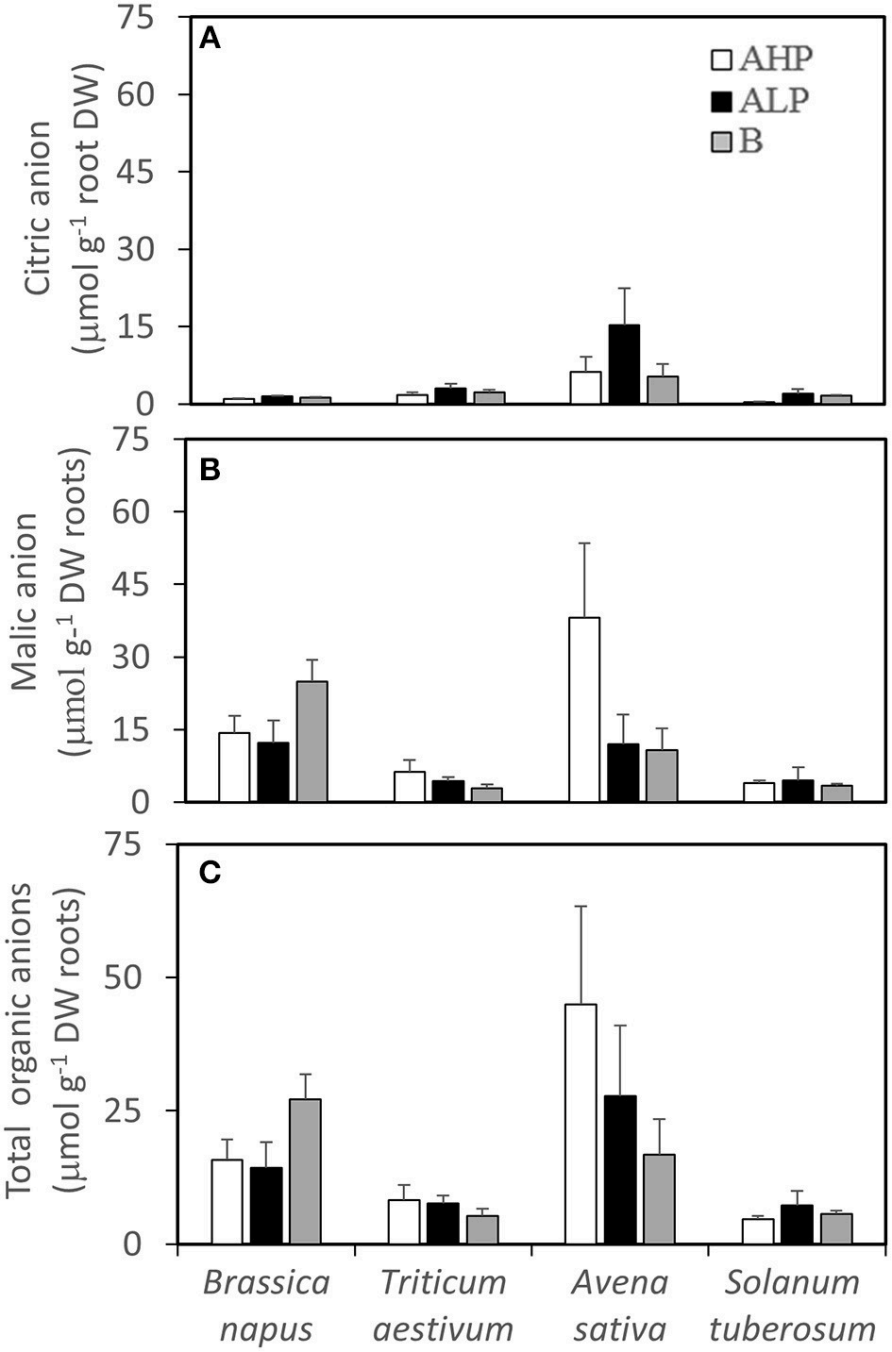
**(A)** Rhizosphere citrate concentrations, **(B)** rhizosphere malate concentrations, and **(C)** rhizosphere total organic anion concentrations of *Brassica napus, Triticum aestivum, Avena sativa*, and *Solanum tuberosum* grown in soils A_HP_ (white bars), A_LP_ (black bars), and B (gray bars) based on root dry weight. Error bars indicate SE (*n* = 4). There was a significant interaction between species and soils for rhizosphere malate concentrations (*p* < 0.05, MSD _0.05_ = 11.47 μmol g^−1^ DW roots).

Organic anions accumulated in the rhizosphere varied significantly among species (Table 2 and Figures 4A–C). Malate, citrate, succinate, and tartrate were detected and malate was the dominant organic anion in the present study. *A. sativa* had the greatest rhizosphere citrate concentration in all soils (Figure 4A), as well as malate (Figure 4B), and total organic anions (Figure 4C) in soil A_HP_, compared with the other three species. In soil B, *B. napus* had the greatest rhizosphere malate and total organic anion concentrations of all the studied species. Generally, more rhizosphere citrate was detected in low P soils than in high P soil while a contrasting pattern was found for malate except for *B. napus*, which showed an increase of malate in soil B, compared with soils A_HP_ and A_LP_ (by 74 and 103%, respectively). Similar patterns were found if the concentrations were calculated based on rhizosphere soil weight, except for *S. tuberosum* (Figure S2).

In soils A_HP_ and A_LP_ (with the same soil texture but different P availability), a significant negative linear correlation was found between the amount of citrate in the rhizosphere and the shoot P concentration for *B. napus* (*r* = −0.82, *p* < 0.01) and *S. tuberosum* (*r* = −0.71, *p* < 0.05). However, a positive correlation was found between the amount of rhizosphere malate and shoot P concentration for *S. tuberosum* (*r* = 0.83, *P* < 0.01, *n* = 8).

### Rhizosphere pH, plant available P, and water-soluble P response

**Figure 5:**
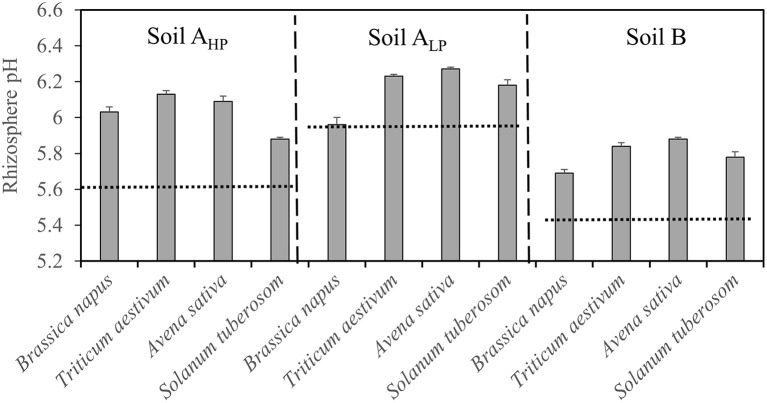
**Rhizosphere soil pH of ***Brassica napus***, ***Triticum aestivum***, ***Avena sativa***, and ***Solanum tuberosum*** grown in soils A_**HP**_, A_**LP**_, and B**. Error bars indicate SE (*n* = 4). There was a significant interaction between species and soils for rhizosphere pH (*p* < 0.001, MSD _0.05_ = 0.1). Dashed lines indicate the soil pH values in unplanted mini-rhizotrons.

The pH of the rhizosphere varied significantly among species and soils (Table 2 and Figure 5). All species generally had a rhizosphere water extract pH between 5.5 and 6.3. In soil A_HP_, *T. aestivum* had the highest rhizosphere pH (6.1) while *S. tuberosum* had the lowest pH (5.9). In soil A_LP_ and soil B, *A. sativa*, followed by *T. aestivum*, had the highest rhizosphere pH and *B. napus* had the lowest pH.

**Figure 6:**
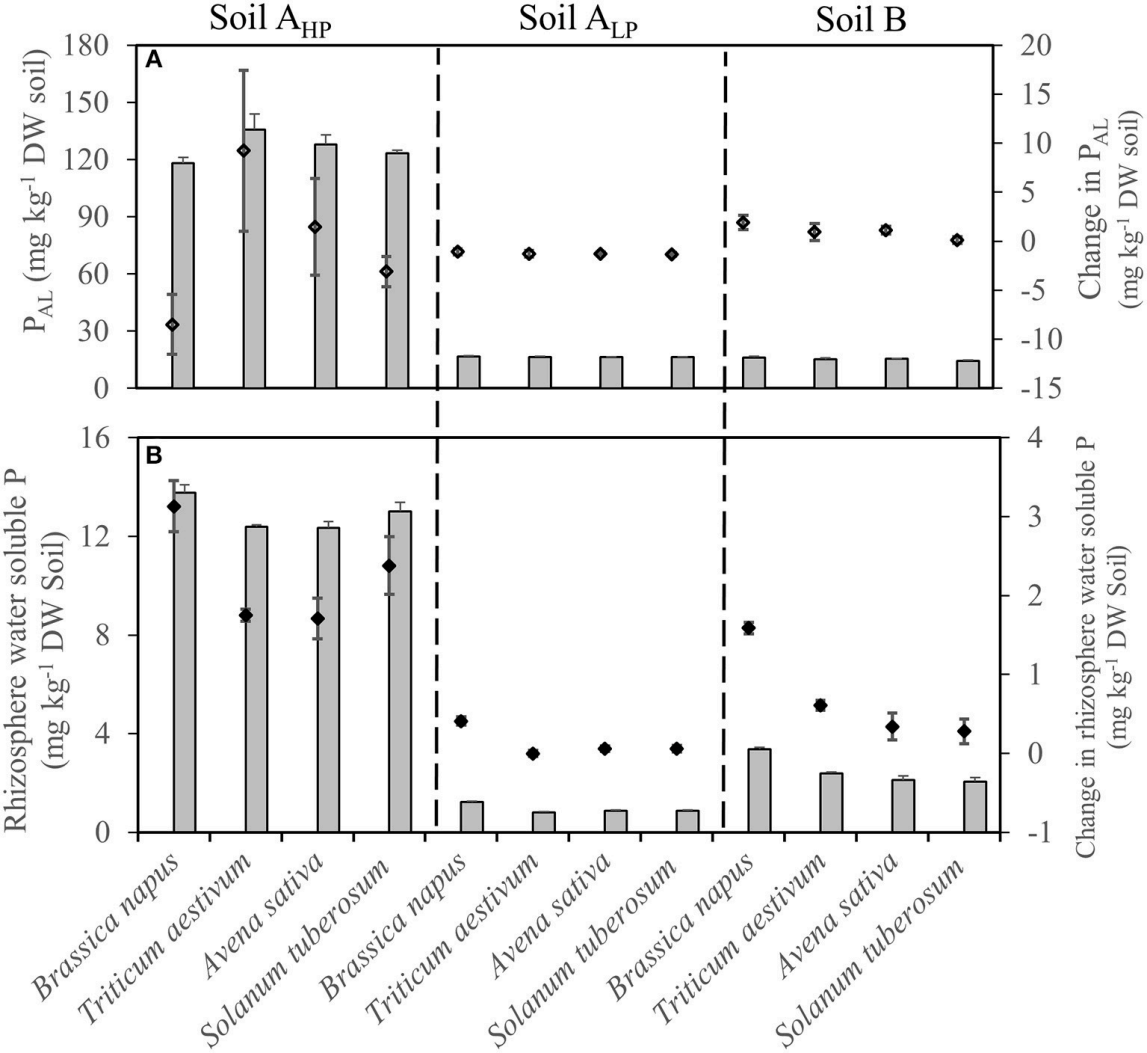
**(A)** Rhizosphere AL-extractable P and **(B)** rhizosphere water soluble P of *Brassica napus, Triticum aestivum, Avena sativa*, and *Solanum tuberosum* grown in soils A_HP_, A_LP_, and B (gray bars), as well as changes compared to unplanted bulk soils (black diamonds). Error bars indicate SE (*n* = 4). There was a significant interaction between species and soils for change in P_AL_ (*p* < 0.05, MSD _0.05_ = 5.63 mg kg^−1^), and change in rhizosphere water soluble P (*p* < 0.01, MSD _0.05_ = 0.34 mg kg^−1^).

The plant-available P (determined as P_AL_) in the rhizosphere increased slightly in soil B but decreased in soil A_LP_ in all species, compared with bulk soils (Figure 6A). The P_AL_ of *T. aestivum* rhizosphere increased slightly in soil A_HP_, while decreasing slightly for *B. napus*.

Water-soluble P (WSP) is the most easily available P for plants. In the present study, almost all the rhizosphere WSP concentrations increased, compared with unplanted bulk soils. However, rhizosphere WSP varied significantly among soils and species (Table 2 and Figure 6B). For the bulk soils, soil A_HP_ had the highest WSP concentrations (10.64 mg kg^−1^ DW soil), and the lowest WSP concentrations were observed in soil A_LP_ (0.81 mg kg^−1^ DW soil). In all the three soils, *B. napus* showed the highest rhizosphere WSP concentrations compared with the other three species. Compared with the unplanted bulk soils, it increased by 3.13 mg kg^−1^ DW soil (29.4%), 0.41 mg kg^−1^ DW soil (50.6%), and 1.59 mg kg^−1^ DW soil (89.3%) in soils A_HP_, A_LP_, and B, respectively (Figure 6B).

### Soil enzyme activities

**Figure 7:**
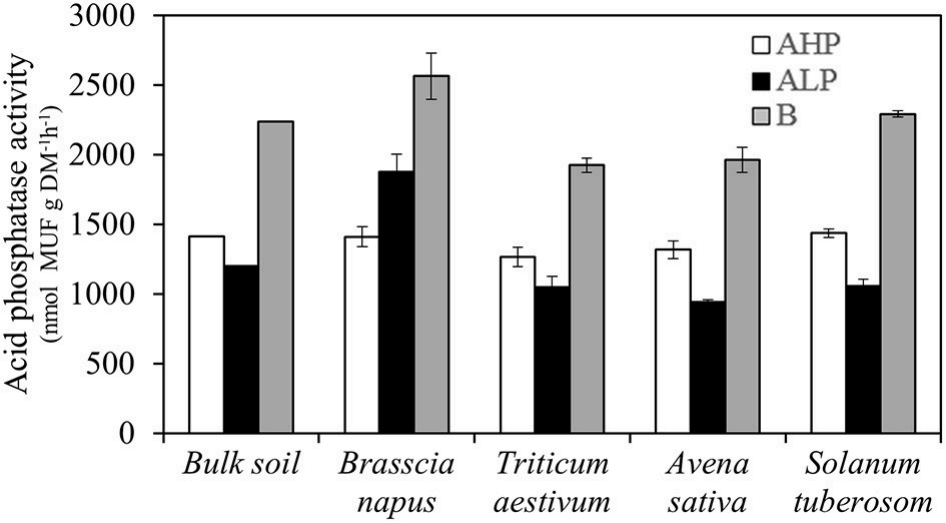
**Acid phosphatase activities of bulk soil, and rhizosphere soil for ***Brassica napus***, ***Triticum aestivum***, ***Avena sativa***, and ***Solanum tuberosum*** grown in soils A_**HP**_ (white bars), A_**LP**_ (black bars), and B (gray bars)**. Error bars indicate SE (*n* = 4). There was a significant interaction between species and soils for acid phosphatase activities (*p* < 0.001, MSD _0.05_ = 153 nmol MUF g^−1^ DM^−1^h^−1^).

Rhizosphere acid phosphatase activity (APase) was not affected by plant species when grown in the soil A_HP_, while in the low P soils, A_LP_ and B, *B. napus* rhizosphere soil had the highest APase activity (Table 2 and Figure 7). The other three enzymes measured in the present study (β-glucosidase, β-xylosidase, and N-acetyl-β-glucosaminidase) were mainly influenced by soil type (Figure S3). Moreover, *B. napus* rhizosphere soil had greater ratios of APase to the other three enzymes in soil A_LP_ than other soils and species (Figure S4).

### Soil microbial biomass P

**Figure 8:**
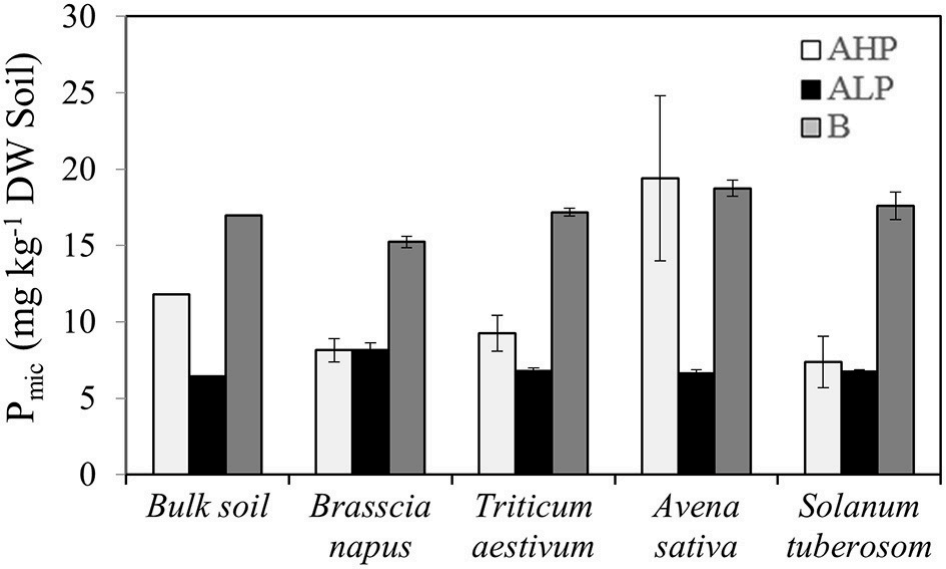
**Phosphorus content immobilized by microbial biomass (P_**mic**_) of bulk soil, and rhizosphere soil for ***Brassica napus***, ***Triticum aestivum***, ***Avena sativa***, and ***Solanum tuberosum*** grown in soils A_**HP**_ (white bars), A_**LP**_ (black bars), and B (gray bars)**. Error bars indicate SE (*n* = 4). There was a significant interaction between species and soils for P_mic_ (*p* < 0.01, MSD _0.05_ = 3.28 mg kg^−1^ DW soil).

The soil microbial biomass P content (P_mic_) was mainly affected by soil type (Table 2 and Figure 8) but showed an interaction with plant species. For bulk soils, P_mic_ declined in the order soil B > A_HP_ > A_LP_. In the plant rhizosphere, soil A_LP_ showed always the lowest and B the highest P_mic_ (except for *A. sativa*). The dicots (*S. tuberosum* and *B. napus*) supported a similar low amount of microbial P when growing in soils A_LP_ and A_HP_, whereas under cereals, soil A_HP_ supported a higher P_mic_ than A_LP_. In soils A_HP_ and B, *A. sativa* had the greatest rhizosphere P_mic_. No significant difference was found for plants grown in soil A_LP_. In addition, *B. napus* rhizosphere soil had greater ratios of APase to P_mic_ in soil A_LP_ than other soils and species (Figure S4).

### AMF and Non-AMF root colonization in mycorrhizal species

For mycorrhizal plant species, non-AMF were more abundant than AMF in roots (Table 3). Neither non-AMF nor AMF were influenced by soil P availability for any of the three species. The percentage of root length colonized differed among the plant species in all soils. *A. sativa* had the highest colonization of both AMF (32–40%) and non-AMF (58–75%) and colonization was lowest in *S. tuberosum* (22–36% of non-AMF and no AMF were detected).

**Table 3:** **The percentage of root length colonized by arbuscular mycorrhizal fungi (AMF) and non-arbuscular mycorrhizal fungi (non-AMF) in mycorrhizal species**.

	**Soil A**_**HP**_		**Soil A**_**LP**_		**Soil B**	
	**AMF**	**Non-AMF**	**AMF**	**Non-AMF**	**AMF**	**Non-AMF**
*Triticum aestivum*	9.6 ± 7.6	46 ± 6.0	11.3 ± 6.1	44 ± 5.3	12.7 ± 5.0	53.3 ± 8.1
*Avena sativa*	32.7 ± 6.0	65.3 ± 6.1	36 ± 12.5	69.3 ± 11.4	40 ± 5.3	74.7 ± 7.0
*Solanum tuberosum*	ND	26.7 ± 5.0	ND	30 ± 6.5	ND	36 ± 5.0

*Values are means ± SE of n = 3–4. ND, not detected*.

### Linear regression analysis

In within-soil analyses, significant correlations were found in low P availability soils A_LP_ and B (Table 4). Plant shoot P content showed strong correlations with rhizosphere water-soluble P, rhizosphere pH, and APase. Total P uptake had weak positive correlations with rhizosphere APase and water-soluble P in soil A_LP_. Moreover, shoot P content and total P content showed strong positive correlations with root mass ratio in soil B. Positive correlations between rhizosphere citrate concentration and root P content were found in soil A_LP_ and soil B and rhizosphere malate correlated weakly with root P content in soil A_LP_. In addition, rhizosphere water-soluble P showed strong correlations with rhizosphere APase and pH. A weak correlation between rhizosphere malate concentration and water-soluble P was also found in soil B. Rhizosphere APase correlated negatively with rhizosphere pH.

**Table 4:** **Correlations within low P availability soils A_**LP**_ and B across species**.

**Response variables**	**Explanatory variables**	***r* (A_LP_)**	***r* (B)**
Shoot P content	Rhizosphere APase	0.77^***^	0.72^**^
	Rhizosphere WSP	0.79^***^	0.85^***^
	Rhizosphere pH	−0.65^**^	−0.79^***^
	Root mass ratio	ns	0.68^***^
Root P content	Rhizosphere citrate	0.62^**^	0.77^***^
	Rhizosphere malate	0.51^*^	ns
Total P content	Rhizosphere WSP	0.59^**^	ns
	Rhizosphere APase	0.56^*^	ns
	Root mass ratio	ns	0.69^**^
Rhizosphere WSP	Rhizosphere APase	0.89^***^	0.59^*^
	Rhizosphere pH	−0.91^***^	−0.75^***^
	Rhizosphere malate	ns	0.55^*^
Rhizosphere APase	Rhizosphere pH	−0.88^***^	−0.76^***^

WSP, water soluble P; r, correlation efficient; ns, not significant;

* p < 0.05;

** p < 0.01;

*** *p < 0.001. n = 16 (4 species × 4 replicates) for every correlation*.

As shown in Table 5, within each species across all three soils, plant P uptake had a strong positive correlation with rhizosphere WSP for all species. Rhizosphere citrate, APase, and root mass ratio explained the WSP and P uptake in *B. napus*. In *A. sativa*, P uptake was linked to rhizosphere malate, APase and pH while rhizosphere APase was linked to root mass ratio. For *S. tuberosum*, P mobilization and uptake was mainly explained by rhizosphere citrate and pH. Across low P soils A_LP_ and B, plant P uptake was mostly explained by rhizosphere APase, pH, and rhizosphere WSP (data not shown) for all species. Rhizosphere pH also correlated significantly with rhizosphere APase and WSP for all species. In addition, rhizosphere APase showed significant correlation with root mass ratio in *A. sativa* and *S. tuberosum*. The only significant correlation between total P uptake and root mass ratio was found in *A. sativa*.

**Table 5:** **Correlations within each species**.

**Data source**	**Response variables**	**Explanatory variables**	***r* (*B. n*)**	***r* (*T. a*)**	***r* (*A. s*)**	***r* (*S. t*)**
Across all three soils (*n* = 12)	Total P content	Rhizosphere WSP	0.96^***^	0.89^***^	0.73^**^	0.90^***^
		Rhizosphere citrate	−0.64^*^	ns	ns	−0.63^*^
		Root mass ratio	−0.71^**^	ns	ns	ns
		Rhizosphere pH	ns	ns	ns	−0.65^*^
	Shoot P content	Rhizosphere malate	ns	ns	0.67^*^	ns
		Rhizosphere pH	ns	ns	ns	−0.64^*^
		Rhizosphere APase	−0.60^*^	ns	ns	ns
		Root mass ratio	−0.79^**^	ns	ns	ns
		Rhizosphere WSP	0.94^***^	0.94^***^	0.95^***^	0.86^***^
	Root P content	Rhizosphere citrate	−0.72^*^	ns	ns	−0.65^*^
		Rhizosphere pH	ns	ns	−0.82^**^	−0.62^*^
		Rhizosphere APase	ns	ns	0.81^**^	ns
		Rhizosphere WSP	0.84^***^	0.68^*^	ns	0.89^***^
	Rhizosphere WSP	Rhizosphere citrate	−0.63^*^	ns	ns	−0.68^*^
		Rhizosphere APase	−0.64^*^	ns	ns	ns
		Root mass ratio	−0.75^**^	ns	−0.60^*^	ns
	Rhizosphere APase	Rhizosphere pH	−0.80^**^	−0.98^***^	−0.95^***^	−0.86^***^
		Root mass ratio	0.68^*^	ns	0.65^*^	ns
Across low P soils A_LP_ and B (*n* = 8)	Total P content	Rhizosphere APase	0.83^**^	0.94^***^	0.91^***^	0.97^***^
		Rhizosphere pH	−0.80^**^	−0.91^***^	−0.89^***^	−0.95^***^
		Root mass ratio	ns	ns	0.77^**^	ns
	Shoot P content	Rhizosphere pH	−0.72^*^	−0.89^***^	ns	−0.94^***^
		Rhizosphere APase	0.77^**^	0.93^***^	ns	0.93^***^
	Root P content	Rhizosphere APase	0.84^**^	ns	0.91^***^	0.94^***^
		Rhizosphere pH	−0.84^**^	ns	−0.91^***^	−0.90^***^
	Rhizosphere WSP	Rhizosphere APase	0.84^**^	0.99^***^	0.92^***^	0.97^***^
		Rhizosphere pH	−0.95^***^	−0.98^***^	−0.96^***^	−0.98^***^
	Rhizosphere APase	Rhizosphere pH	−0.70^*^	−0.98^***^	−0.98^***^	−0.99^***^
		Root mass ratio	ns	ns	0.83^*^	0.69^*^

B. n, T. a, A. s, and S. t represent Brassica napus, Triticum aestivum, Avena sativa, and Solanum tuberosum respectively; WSP, water soluble P; r, correlation efficient; ns, not significant;

* p < 0.05;

** p < 0.01;

*** *p < 0.001*.

## Discussion

The results of this experiment suggested that rhizosphere organic anions made a minor contribution to P mobilization and uptake for the studied crops in the studied low P clay loam and loam soils. Different plant species may have different growth potential in a mini-rhizotron and different needs for P to support their development. In addition to plant species, rhizosphere WSP, APase, and pH were likely to affect P availability and uptake. Unsurprisingly, rhizosphere WSP appears to have contributed greatly to P uptake in both high and low P availability soils. *B. napus* took up P efficiently, possibly due to greater rhizosphere APase activities and lower rhizosphere pH. *A. sativa* was another crop that could use P efficiently in our study, possibly due to its greater root mass ratio and higher percentage of root colonizing AMF. *A. sativa* had a larger proportion of root length colonized by AMF than *T. aestivum* and *S. tuberosum*, which might benefit its P acquisition. The implications of these findings and other points of interest are discussed below.

### Effects of rhizosphere organic anions on soil pH, water soluble P and P uptake

When studying rhizosphere organic anions, the great challenge is how to extract them effectively from soil samples due to their ability to interact with soil particles (Valentinuzzi et al., 2015). Low concentrations of Ca are often added to the extractant to ensure cell integrity, limit osmotic stress, and possible leakage/diffusion. Although Micropur can inhibit organic anion degradation by microorganisms, it also influences the exudation and degradation processes and hence affects the results (Valentinuzzi et al., 2015). Moreover, root exudates have complicated interactions with soil microbial communities (Badri and Vivanco, 2009). Therefore, it is very hard to extract all the rhizosphere organic anions and to perform accurate analytical determination. In addition, not all the rhizosphere soil in this study was taken to extract organic anions due to other soil measurements. Taking all the above factors into account, our data probably underestimated organic anion concentrations but may still reflect the relative differences between the plant species.

Generally, more citrate was detected in A_LP_ than in A_HP_ while the opposite was the case for malate for all species, and more total organic anions were detected in A_LP_ than A_HP_ only for *S. tuberosum*. Hence, our hypothesis (1) that low P availability stimulates plant roots to release more organic anions to the soil was not fully supported in our system. Inconsistent with our earlier results using hydroponic culture (Wang et al., 2015), we detected citrate in *S. tuberosum* rhizosphere, which may be due to longer-term accumulation in the soil experiment while we collected root exudates for only 2 h in the hydroponic system; alternatively, it may have been produced by soil microbes. We found that *B. napus* had higher rhizosphere total organic anion concentrations than *T. aestivum* and *S. tuberosum* but lower than those of *A. sativa*. Considering that *A. sativa* also showed highest AMF colonization and rhizosphere P_mic_, these soil microbes might explain large amounts of the rhizosphere organic anions found. AMF-released carbon can trigger phosphate-solubilizing bacterium growth and activity (Zhang et al., 2016) and these bacteria can release organic anions to the soil (Jones, 1998). In addition, in soils A_HP_ and A_LP_ (soils with the same soil texture but different P availabilities), shoot P concentrations of *B. napus* and *S. tuberosum* had negative correlations with rhizosphere citrate concentration. Therefore, our hypothesis (3) was supported.

Root-released organic anions have been widely documented as a key physiological strategy to mobilize P from plant less-available P sources (e.g., Raghothama, 1999; Lambers et al., 2006, 2015). In our study, no simple linear relationships were found between rhizosphere organic anions and WSP, except that in soil B, malate concentration had weak correlation with rhizosphere WSP. Within species, *B. napus* and *S. tuberosum* showed weak negative correlations between total P content, root P content and rhizosphere WSP with citrate across the three soils but not in low P soils A_LP_ and B. These correlations might therefore be due to the influence of data from high P availability soil A_HP_. We found that the rhizosphere citrate and malate concentrations had weak positive correlations with root P content in low P soils A_LP_ and B, but no correlations with shoot and total P content. This was not consistent with some previous reports which reported strong positive correlations between total P content and rhizosphere organic anions in legumes and 12 *Kennedia* species (Ryan et al., 2012; Pang et al., 2015). These two studies were conducted in river sand to minimize interference of soil with P availability and varyingly soluble P was applied. However, our results compared well with the findings of Nazeri et al. (2014), who used agricultural soil. These findings suggested that rhizosphere organic anions may play a minor role in improving soil P availability and plant P uptake. Although organic acids have often been referred to as a possible source of rhizosphere acidification (Hinsinger, 2001), the effects of organic anion exudation on pH are complex (Roelofs et al., 2001). No correlation between rhizosphere organic anions and rhizosphere pH compared well with previous reports (Pang et al., 2010, 2015) and suggested that other factors were more important in influencing soil pH. A similar result was reported in *B. napus*; P deficiency induced decrease of rhizosphere pH was not associated with the increase of extractable rhizosphere organic anions but mainly from root released H^+^ (Hedley et al., 1982a). Overall, our results suggesting that rhizosphere organic anions make a minor contribution to P uptake agree with other reports (Pearse et al., 2007; Pandey et al., 2014; Ryan et al., 2014). In many cases, a single trait like larger amounts of rhizosphere organic anions did not result in improved P uptake and other factors such as root morphology (which we did not investigate) and pH may play key roles. Hence, hypothesis (2) that rhizosphere organic anions correlate with P availability and uptake was not supported, while hypothesis (3) was partly supported.

### Effects of phosphatase activity on P uptake

Compared with A_HP_, rhizosphere APase activity decreased in A_LP_ for all species except *B. napus*, hence, our hypothesis (1) that low P availability will induce plant roots to release more APase to the soil was rejected. APase increased greatly in soil B compared with A_HP_ and A_LP_ indicating that other soil properties (possibly for example texture) have strong influence on plant rhizosphere APase. Across different crops, plant shoot P uptake, and rhizosphere water-soluble P had a strong positive correlation with rhizosphere APase in low P soils. In addition, total P uptake had a significant correlation with rhizosphere APase across crops in low P soil A_LP_ and within all species across low P soils. Acid phosphatase, which originates from both plants and soil microbes (Lambers et al., 2006), could hydrolyze organic P compounds in soil and increase P availability, thereby enhancing plant P uptake (Tarafdar et al., 2001; George et al., 2004; Richardson et al., 2009). Tarafdar et al. (2001) suggested that microbes are responsible for the dominant contribution to soil phosphatases. This seems to be the case in our study as the bulk soil analysis also showed high APase concentrations. However, some reports have indicated that plant roots have a major impact on the composition and function of the rhizosphere microbial community, and phosphatase in the rhizosphere is mainly secreted by plant roots rather than by microbial activity in response to P limitation (Kandeler et al., 2002; Wasaki et al., 2008). For *B. napus* exposed to P deficiency, rhizosphere APase activity increased 10 times compared with bulk soil and appeared to be a response to increasing root density and no evidence was found for significant hydrolysis of (Hedley et al., 1982b, 1983). We also found that APase had significant negative correlation with rhizosphere WSP and positive correlation with root mass ratio in *B. napus* across all soils. Therefore, compared with the bulk soils, the increase in APase activities in rhizosphere soils of *B. napus* in low P soils might be from the roots. In addition, rhizosphere APase had a strong negative correlation with rhizosphere pH in low P availability soils, which was consistent with previous reports (Mobley et al., 1984; Šarapatka et al., 2004). Taking all of the above together, our hypothesis (2) that APase correlates with P availability and uptake and hypothesis (3) were supported. Further study is needed to prove whether APase can hydrolyze soil organic P.

### Effects of mycorrhizae on plant responses to low P availability

In the present study, both mycorrhizal and non-mycorrhizal species were selected. Significant differences were found for AMF colonization among the three mycorrhizal plant species but this was not affected by soil P availability. Results reported in the literature vary. According to a previous review, high P application can decrease both root and soil AMF biomass per plant (Smith et al., 2011). In a semi-arid grassland experiment, AMF colonization was reduced significantly when soil P was high in 1 year, but not in the following year, probably due to the effects of other environmental factors (Klabi et al., 2015). Moreover, a 15 mg kg^−1^ P pulse treatment did not affect the percentage of root length colonized in five legumes grown in sandy soil with moderate P content of 11 mg kg^−1^ Olsen P (Nazeri et al., 2014). Our results that soil P did not affect AMF colonization are thus in accordance with the second year's results of the semi-arid grassland experiment and also consistent with the report by Nazeri et al. (2014). Nazeri et al. (2014) also reported that inoculation with AMF in five legumes decreased the amount of rhizosphere carboxylates by 52%, raised the rhizosphere pH by 0.2–0.7 pH units and was associated with higher rhizosphere Colwell P (bicarbonate-extractable P). Although our results show that *A. sativa*, which had a higher coverage percent of root colonizing AMF, had higher rhizosphere pH and lower APase in low P soils than the non-mycorrhizal plant *B. napus*, we could not reach any conclusions due to lack of non-mycorrhizal control treatments and enough non-mycorrhizal species. It would be necessary to carry out new experiments that include proper non-mycorrhizal control to reveal the mechanisms involved.

### Other factors possibly involved in P uptake

In our study, WSP correlated highly with P uptake. Hence, WSP can be used to evaluate a soil's P availability, as well as P_AL_, Olsen P, and Colwell P. We observed an increase in rhizosphere WSP, which is different from other reports which showed decreases in rhizosphere Olsen P or Colwell P during plant growth (Nazeri et al., 2014; Li et al., 2016). Therefore, WSP can be used as a parameter to study P mobilization. Root morphology is also a key factor that affects P uptake (Lambers et al., 2006, 2008; Lynch, 2011; Pedas et al., 2011). We measured root morphology in our previous experiment (Wang et al., 2015) but not in this study so it will not be discussed here. Rhizosphere pH is another important factor involved in this study, lower rhizosphere pH may result in higher APase activity (Mobley et al., 1984; Šarapatka et al., 2004) and altered P sorption/desorption (Singh et al., 2005), thereby affecting P uptake through changing plant available P. Indeed, the rhizosphere pH decreased almost 2.4 units after 2 weeks and was raised slightly after 5 weeks when *B. napus* was grown in thin layers of a P-deficient soil, and the pH decrease was associated with an increase in rhizosphere available P (Grinsted et al., 1982; Hedley et al., 1982b). We found significant correlations of pH with WSP and APase within or across species in low P soils.

Moreover, it has been reported that phosphorus adsorption by soil was enhanced with an increase in clay content in suspension (Syers et al., 1973; Ullah et al., 1983), since with increasing clay content there is often increased content of Fe- and Al-(hydro)oxides, which are important constituents for P sorption. A lower content of clay in soil B suggests that this soil might adsorb less P (lower P sorption capacity) and organic anions than soil A_LP_ and that plants may therefore grow better in soil B than in soil A_LP_. Another explanation is that more sand in soil B makes it more porous, which probably gives better aeration of the soil and thereby more oxygen to the roots. Further studies in long-term field experiments under varied agricultural soil textures might help to clarify this.

## Conclusions

Through this experiment using four common crops and three agricultural soils, we found that plant P uptake may be linked to rhizosphere WSP, APase activity, and pH. Rhizosphere organic anions appear to play a minor role in improving P uptake. We conclude that our hypothesis (1) that low P availability soils will stimulate plant roots to release more organic anions and phosphatase enzymes to rhizosphere soil was not supported. Our hypothesis (2) that the amounts of rhizosphere APase will have positive correlations with rhizosphere plant-available P fractions and P uptake by plants in low P soils was supported, but this was not the case for rhizosphere organic anions. WSP can be used to study P mobilization and assess soil P availability. Hypothesis (3) that different crops will show differing root released organic anions and APase in terms of using residual P from agricultural soils was supported. We found that both *B. napus* and *A. sativa* are good candidates to study P utilization. The results and information generated in this study are valuable for understanding P mobilization and P uptake in low P agricultural soils, and for future effective utilization of P and improving the productivity of the studied crops.

## Author contributions

NC, TK, JC, AØ, SE, and YW made contributions to the design of the study. YW conducted the experiment, collected and analyzed the samples, and drafted the manuscript. MH and EK made a contribution to analysis of soil enzyme activities and P_mic_. All authors participated in preparing the manuscript.

## Funding

This study was supported by the strategic institute program on “Opportunities for sustainable use of phosphorus in food production” at the Norwegian Institute of Bioeconomy Research.

### Conflict of interest statement

The authors declare that the research was conducted in the absence of any commercial or financial relationships that could be construed as a potential conflict of interest. The reviewer IJ and handling Editor declared their shared affiliation, and the handling Editor states that the process nevertheless met the standards of a fair and objective review.

## Supplementary material

Figure S1 **(A)** Shoot P content and **(B)** root P content of *Brassica napus, Triticum aestivum, Avena sativa* and *Solanum tuberosum* grown in soils A_HP_ (stippled bars), A_LP_ (black bars), and B (gray bars). Error bars indicate SE (*n* = 4).

Figure S2 **(A)** Rhizosphere citrate concentrations, **(B)** rhizosphere malate concentrations, and **(C)** rhizosphere total organic anion concentrations for *Brassica napus, Triticum aestivum, Avena sativa*, and *Solanum tuberosum* grown in soils A_HP_ (white bars), A_LP_ (black bars), and B (gray bars) based on rhizosphere soil dry weight. Error bars indicate SE (*n* = 4).

Figure S3 **(A)** β-glucosidase activity, **(B)** β-xylosidase activity, and **(C)** N-acetyl-β-glucosaminidase activity of bulk soil for *Brassica napus, Triticum aestivum, Avena sativa*, and *Solanum tuberosum* grown in soils A_HP_ (white bars), A_LP_ (black bars) and B (gray bars). Error bars indicate SE (*n* = 4).

Figure S4 **(A)** Ratio of acid phosphatase activity to P_mic_, **(B)** ratio of acid phosphatase activity to β-glucosidase activity, **(C)** ratio of acid phosphatase activity to N-acetyl-β-glucosaminidase activity, and **(D)** ratio of acid phosphatase activity to β-xylosidase activity of bulk soil, for *Brassica napus, Triticum aestivum, Avena sativa*, and *Solanum tuberosum* grown in soils A_HP_ (white bars), A_LP_ (black bars), and B (gray bars). Error bars indicate SE (*n* = 4).

## References

[B1] Badri D. V. Vivanco J. M. (2009). Regulation and function of root exudates. Plant Cell Environ. 32, 666–681. 10.1111/j.1365-3040.2009.01926.x 19143988

[B2] Cheng L. Tang X. Vance C. P. White P. J. Zhang F. Shen J. (2014). Interactions between light intensity and phosphorus nutrition affect the phosphate-mining capacity of white lupin (*Lupinus albus* L.). J. Exp. Bot. 65, 2995–3003. 10.1093/jxb/eru135 24723402 PMC4071820

[B3] Egnér H. Riehm H. Domingo W. (1960). Untersuchungen über die chemische Bodenanalyse als Grundlage für die Beurteilung des Nährstoffzustandes der Böden. II. Chemische Extraktionsmethoden zur Phosphor-und Kaliumbestimmung. Kungliga Lantbrukshögskolans Annaler 26, 199–215.

[B4] Faucon M.-P. Houben D. Reynoird J.-P. Mercadal-Dulaurent A.-M. Armand R. Lambers H. (2015). Advances and perspectives to improve the phosphorus availability in cropping systems for agroecological phosphorus management. Adv. Agron. 134, 51–79. 10.1016/bs.agron.2015.06.003

[B5] Gahoonia T. S. Asmar F. Giese H. Gissel-Nielsen G. Nielsen N. E. (2000). Root-released organic acids and phosphorus uptake of two barley cultivars in laboratory and field experiments. Eur. J. Agron. 12, 281–289. 10.1016/S1161-0301(00)00052-6

[B6] George T. Richardson A. Hadobas P. Simpson R. (2004). Characterization of transgenic *Trifolium subterraneum* L. which expresses phyA and releases extracellular phytase: growth and P nutrition in laboratory media and soil. Plant Cell Environ. 27, 1351–1361. 10.1111/j.1365-3040.2004.01225.x

[B7] German D. P. Weintraub M. N. Grandy A. S. Lauber C. L. Rinkes Z. L. Allison S. D. (2011). Optimization of hydrolytic and oxidative enzyme methods for ecosystem studies. Soil Biol. Biochem. 43, 1387–1397. 10.1016/j.soilbio.2011.03.017

[B8] Giacometti C. Cavani L. Baldoni G. Ciavatta C. Marzadori C. Kandeler E. (2014). Microplate-scale fluorometric soil enzyme assays as tools to assess soil quality in a long-term agricultural field experiment. Appl. Soil Ecol. 75, 80–85. 10.1016/j.apsoil.2013.10.009

[B9] Giovanetti M. Mosse B. (1980). An evaluation of techniques for measuring vesicular arbuscular mycorrhizal infection in roots. New Phytol. 84, 489–500. 10.1111/j.1469-8137.1980.tb04556.x

[B10] Grinsted M. Hedley M. White R. Nye P. (1982). Plant-induced changes in the rhizosphere of rape (*Brassica napus* var. Emerald) seedlings. I. pH change and the increase in P concentration in the soil solution. New Phytol. 91, 19–29. 10.1111/j.1469-8137.1982.tb03289.x

[B11] Hedley M. J. Nye P. H. White R. E. (1982a). Plant-induced changes in the rhizosphere of rape (*Brassica napus* var. Emerald) seedlings. II. Origin of the pH change. New Phytol. 91, 31–44.

[B12] Hedley M. J. White R. E. Nye P. H. (1982b). Plant-induced changes in the rhizosphere of rape (*Brassica napus* var. Emerald) seedlings. III. Changes in L value, soil phosphate fractions and phosphatase activity. New Phytol. 91, 45–56.

[B13] Hedley M. J. White R. E. Nye P. H. (1983). Plant-induced changes in the rhizosphere of rape (*Brassica napus* var. Emerald) seedlings. IV. The effect of rhizosphere phosphorus status on the pH, phosphatase activity and depletion of soil phosphorus fractions in the rhizosphere and on the cation-anion balance in the plants. New Phytol. 95, 69–82.

[B14] Hinsinger P. (2001). Bioavailability of soil inorganic P in the rhizosphere as affected by root-induced chemical changes: a review. Plant Soil 237, 173–195. 10.1023/A:1013351617532

[B15] Hoffland E. Findenegg G. R. Nelemans J. A. (1989). Solubilization of rock phosphate by rape. Plant Soil 113, 155–160. 10.1007/BF02280175

[B16] James B. R. Bartlett R. J. Amadon J. F. (1985). A root observation and sampling chamber for pot studies. Plant Soil 85, 291–293. 10.1007/BF02139633

[B17] Jones D. L. (1998). Organic acids in the rhizosphere–a critical review. Plant Soil 205, 25–44. 10.1023/A:1004356007312

[B18] Kandeler E. Marschner P. Tscherko D. Gahoonia T. Nielsen N. (2002). Structural and functional diversity of soil microbial community in the rhizosphere of maize. Plant Soil 238, 301–312. 10.1023/A:1014479220689

[B19] Klabi R. Bell T. H. Hamel C. Iwaasa A. Schellenberg M. Raies A.. (2015). Plant assemblage composition and soil P concentration differentially affect communities of AM and total fungi in a semi-arid grassland. FEMS Microbiol. Ecol. 91, 1–13. 10.1093/femsec/fiu015 25764537

[B20] Kouno K. Tuchiya Y. Ando T. (1995). Measurement of soil microbial biomass phosphorus by an anion exchange membrane method. Soil Biol. Biochem. 27, 1353–1357. 10.1016/0038-0717(95)00057-L

[B21] Kristoffersen A. Ø. Riley H. (2005). Effects of soil compaction and moisture regime on the root and shoot growth and phosphorus uptake of barley plants growing on soils with varying phosphorus status. Nutr. Cycl. Agroecosyst. 72, 135–146. 10.1007/s10705-005-0240-8

[B22] Krogstad T. Øgaard A. F. Kristoffersen A. Ø. (2008). New PRecommendations for Grass and Cereals in Norwegian Agriculture. NJF Rep. 4, 42–46.

[B23] Lambers H. Hayes P. E. Laliberté E. Oliveira R. S. Turner B. L. (2015). Leaf manganese accumulation and phosphorus-acquisition efficiency. Trends Plant Sci. 20, 83–90. 10.1016/j.tplants.2014.10.007 25466977

[B24] Lambers H. Raven J. A. Shaver G. R. Smith S. E. (2008). Plant nutrient-acquisition strategies change with soil age. Trends Ecol. Evol. 23, 95–103. 10.1016/j.tree.2007.10.008 18191280

[B25] Lambers H. Shane M. W. Cramer M. D. Pearse S. J. Veneklaas E. J. (2006). Root structure and functioning for efficient acquisition of phosphorus: matching morphological and physiological traits. Ann. Bot. 98, 693–713. 10.1093/aob/mcl114 16769731 PMC2806175

[B26] Li C. Dong Y. Li H. Shen J. Zhang F. (2016). Shift from complementarity to facilitation on P uptake by intercropped wheat neighboring with faba bean when available soil P is depleted. Sci. Rep. 6:18663. 10.1038/srep18663 26728339 PMC4700499

[B27] Lü J. Gao X. Dong Z. Yi J. An L. (2012). Improved phosphorus acquisition by tobacco through transgenic expression of mitochondrial malate dehydrogenase from Penicillium oxalicum. Plant Cell Rep. 31, 49–56. 10.1007/s00299-011-1138-3 21863348

[B28] Lynch J. P. (2011). Root phenes for enhanced soil exploration and phosphorus acquisition: tools for future crops. Plant Physiol. 156, 1041–1049. 10.1104/pp.111.175414 21610180 PMC3135935

[B29] Maguire R. Foy R. Bailey J. Sims J. (2001). Estimation of the phosphorus sorption capacity of acidic soils in Ireland. Eur. J. Soil Sci. 52, 479–487. 10.1046/j.1365-2389.2001.00394.x

[B30] Mobley D. M. Chengappa M. M. Kadel W. L. Stuart J. G. (1984). Effect of pH, temperature and media on acid and alkaline phosphatase activity in “clinical” and “nonclinical” isolates of *Bordetella bronchiseptica*. Can. J. Comp. Med. 48, 175–178. 6722645 PMC1236033

[B31] Murphy J. Riley J. P. (1962). A modified single solution method for the determination of phosphate in natural waters. Anal. Chim. Acta 27, 31–36. 10.1016/S0003-2670(00)88444-5

[B32] Nazeri N. K. Lambers H. Tibbett M. Ryan M. H. (2014). Moderating mycorrhizas: arbuscular mycorrhizas modify rhizosphere chemistry and maintain plant phosphorus status within narrow boundaries. Plant Cell Environ. 37, 911–921. 10.1111/pce.12207 24112081

[B33] Ogner G. Wickstrøm T. Remedios G. Gjelsvik S. Hensel G. R. Jacobsen J. E. (1999). The Chemical Analysis Program of the Norwegian Forest Research Institute 2000. Ås: Norwegian Forest Research Institute.

[B34] Pandey R. Meena S. K. Krishnapriya V. Ahmad A. Kishora N. (2014). Root carboxylate exudation capacity under phosphorus stress does not improve grain yield in green gram. Plant Cell Rep. 33, 919–928. 10.1007/s00299-014-1570-2 24493254

[B35] Pang J. Ryan M. H. Tibbett M. Cawthray G. R. Siddique K. H. Bolland M. D. (2010). Variation in morphological and physiological parameters in herbaceous perennial legumes in response to phosphorus supply. Plant Soil 331, 241–255. 10.1007/s11104-009-0249-x

[B36] Pang J. Yang J. Lambers H. Tibbett M. Siddique K. H. Ryan M. H. (2015). Physiological and morphological adaptations of herbaceous perennial legumes allow differential access to sources of varyingly soluble phosphate. Physiol. Plant. 154, 511–525. 10.1111/ppl.12297 25291346

[B37] Pearse S. J. Veneklaas E. J. Cawthray G. Bolland M. D. Lambers H. (2007). Carboxylate composition of root exudates does not relate consistently to a crop species' ability to use phosphorus from aluminium, iron or calcium phosphate sources. New Phytol. 173, 181–190. 10.1111/j.1469-8137.2006.01897.x 17176404

[B38] Pedas P. Husted S. Skytte K. Schjoerring J. K. (2011). Elevated phosphorus impedes manganese acquisition by barley plants. Front. Plant Sci. 2:37. 10.3389/fpls.2011.00037 22639592 PMC3355622

[B39] Raghothama K. (1999). Phosphate acquisition. Ann. Rev. Plant Biol. 50, 665–693. 10.1146/annurev.arplant.50.1.665 15012223

[B40] Richardson A. E. Hocking P. J. Simpson R. J. George T. S. (2009). Plant mechanisms to optimise access to soil phosphorus. Crop Pasture Sci. 60, 124–143. 10.1071/CP07125

[B41] Roelofs R. Rengel Z. Cawthray G. Dixon K. Lambers H. (2001). Exudation of carboxylates in Australian *Proteaceae*: chemical composition. Plant Cell Environ. 24, 891–904. 10.1046/j.1365-3040.2001.00741.x

[B42] Ryan M. H. Tibbett M. Edmonds-Tibbett T. Suriyagoda L. D. Lambers H. Cawthray G. R.. (2012). Carbon trading for phosphorus gain: the balance between rhizosphere carboxylates and arbuscular mycorrhizal symbiosis in plant phosphorus acquisition. Plant Cell Environ. 35, 2170–2180. 10.1111/j.1365-3040.2012.02547.x 22632405

[B43] Ryan P. Delhaize E. Jones D. (2001). Function and mechanism of organic anion exudation from plant roots. Ann. Rev. Plant Biol. 52, 527–560. 10.1146/annurev.arplant.52.1.527 11337408

[B44] Ryan P. R. James R. A. Weligama C. Delhaize E. Rattey A. Lewis D. C.. (2014). Can citrate efflux from roots improve phosphorus uptake by plants? Testing the hypothesis with near-isogenic lines of wheat. Physiol. Plant. 151, 230–242. 10.1111/ppl.12150 24433537

[B45] Šarapatka B. Dudová L. Kršková M. (2004). Effect of pH and phosphate supply on acid phosphatase activity in cereal roots. Biol. Bratislava 59, 127–131.

[B46] Singh B. R. Subramaniam V. (1996). Phosphorus supplying capacity of heavily fertilized soils II. Dry matter yield of successive crops and phosphorus uptake at different temperatures. Nutr. Cycl. Agroecosyst. 47, 123–134. 10.1007/BF01991544

[B47] Singh B. R. Krogstad T. Shivay Y. S. Shivakumar B. G. Bakkegard M. (2005). Phosphorus fractionation and sorption in P-enriched soils of Norway. Nutr. Cycl. Agroecosyst. 73, 245–256. 10.1007/s10705-005-2650-z

[B48] Smith S. E. Jakobsen I. Grønlund M. Smith F. A. (2011). Roles of arbuscular mycorrhizas in plant phosphorus nutrition: interactions between pathways of phosphorus uptake in arbuscular mycorrhizal roots have important implications for understanding and manipulating plant phosphorus acquisition. Plant Physiol. 156, 1050–1057. 10.1104/pp.111.174581 21467213 PMC3135927

[B49] Smith S. E. Read D. J. (2010). Mycorrhizal Symbiosis. London: Academic Press.

[B50] Smith S. E. Smith F. A. Jakobsen I. (2003). Mycorrhizal fungi can dominate phosphate supply to plants irrespective of growth responses. Plant Physiol. 133, 16–20. 10.1104/pp.103.024380 12970469 PMC1540331

[B51] Syers J. Browman M. Smillie G. Corey R. (1973). Phosphate sorption by soils evaluated by the Langmuir adsorption equation. Soil Sci. Soc. Am. J. 37, 358–363. 10.2136/sssaj1973.03615995003700030015x

[B52] Tarafdar J. C. Yadav R. S. Meena S. C. (2001). Comparative efficiency of acid phosphatase originated from plant and fungal sources. J. Plant Nutr. Soil Sci. 164, 279–282. 10.1002/1522-2624(200106)164:3<279::AID-JPLN279>3.0.CO;2-L

[B53] Ullah M. Jabbar A. Khan M. (1983). The influence of soil pH and texture on the adsorption of phosphorus by soils. Pakistan J. Agr. Res. 4, 41–46.

[B54] Valentinuzzi F. Cesco S. Tomasi N. Mimmo T. (2015). Influence of different trap solutions on the determination of root exudates in *Lupinus albus* L. Biol. Fertil. Soils 51, 757–765. 10.1007/s00374-015-1015-2

[B55] Vance C. P. Uhde-Stone C. Allan D. L. (2003). Phosphorus acquisition and use: critical adaptations by plants for securing a nonrenewable resource. New Phytol. 157, 423–447. 10.1046/j.1469-8137.2003.00695.x 33873400

[B56] van Duivenbooden N. de Wit C. T. van Keulen H. (1995). Nitrogen, phosphorus and potassium relations in five major cereals reviewed in respect to fertilizer recommendations using simulation modelling. Fert. Res. 44, 37–49. 10.1007/BF00750691

[B57] Veneklaas E. J. Stevens J. Cawthray G. R. Turner S. Grigg A. M. Lambers H. (2003). Chickpea and white lupin rhizosphere carboxylates vary with soil properties and enhance phosphorus uptake. Plant Soil 248, 187–197. 10.1023/A:1022367312851

[B58] Vierheilig H. Coughlan A. P. Wyss U. Piché Y. (1998). Ink and vinegar, a simple staining technique for arbuscular-mycorrhizal fungi. Appl. Environ. Microbiol. 64, 5004–5007. 9835596 10.1128/aem.64.12.5004-5007.1998 PMC90956

[B59] Wang Y.-L. Almvik M. Clarke N. Eich-Greatorex S. Øgaard A. F. Krogstad T.. (2015). Contrasting responses of root morphology and root-exuded organic acids to low phosphorus availability in three important food crops with divergent root traits. AoB Plants 7:plv097. 10.1093/aobpla/plv097 26286222 PMC4583607

[B60] Wang Y. Xu H. Kou J. Shi L. Zhang C. Xu F. (2013). Dual effects of transgenic *Brassica napus* overexpressing CS gene on tolerances to aluminum toxicity and phosphorus deficiency. Plant Soil 362, 231–246. 10.1007/s11104-012-1289-1

[B61] Wasaki J. Kojima S. Maruyama H. Haase S. Osaki M. Kandeler E. (2008). Localization of acid phosphatase activities in the roots of white lupin plants grown under phosphorus-deficient conditions. Soil Sci. Plant Nutr. 54, 95–102. 10.1111/j.1747-0765.2007.00207.x

[B62] Watts-Williams S. J. Smith F. A. McLaughlin M. J. Patti A. F. Cavagnaro T. R. (2015). How important is the mycorrhizal pathway for plant Zn uptake? Plant Soil 390, 157–166. 10.1007/s11104-014-2374-4

[B63] Zhang L. Xu M. Liu Y. Zhang F. Hodge A. Feng G. (2016). Carbon and phosphorus exchange may enable cooperation between an arbuscular mycorrhizal fungus and a phosphate-solubilizing bacterium. New Phytol. 210, 1022–1032. 10.1111/nph.13838 27074400

