# Supplementary figures and images for: Rhizosphere Organic Anions Play a Minor Role in Improving Crop Species' Ability to Take Up Residual Phosphorus (P) in Agricultural Soils Low in P Availability
Source: Front Plant Sci. 2016 Nov 7;7:1664. doi: 10.3389/fpls.2016.01664 (PMC5097927; doi:10.3389/fpls.2016.01664)

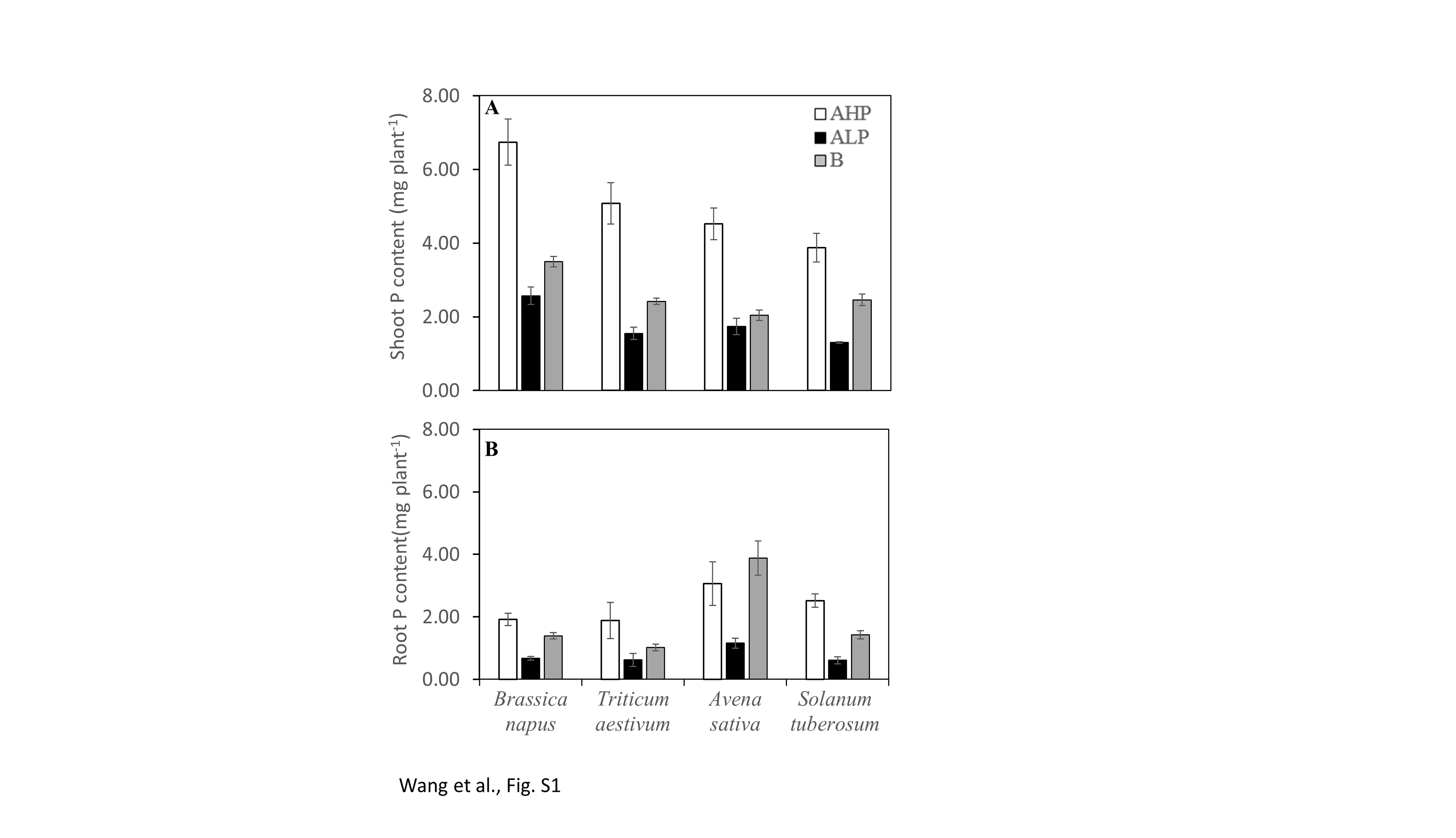

Supplement: Figure S1 — (A) Shoot P content and (B) root P content of Brassica napus, Triticum aestivum, Avena sativa and Solanum tuberosum grown in soils AHP (stippled bars), ALP (black bars), and B (gray bars). Error bars indicate SE (n = 4). [file Image1.tif]

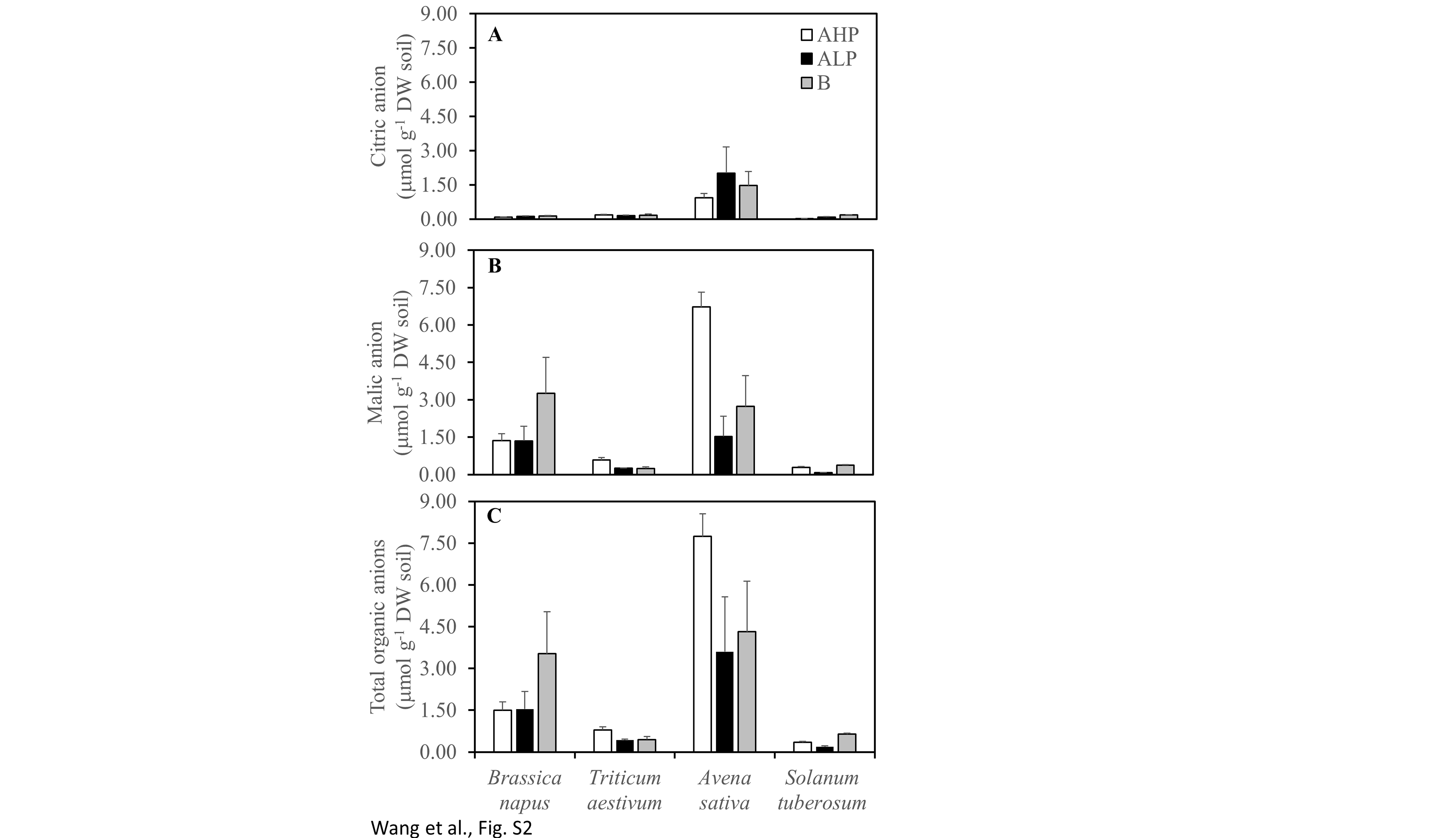

Supplement: Figure S2 — (A) Rhizosphere citrate concentrations, (B) rhizosphere malate concentrations, and (C) rhizosphere total organic anion concentrations for Brassica napus, Triticum aestivum, Avena sativa, and Solanum tuberosum grown in soils AHP (white bars), ALP (black bars), and B (gray bars) based on rhizosphere soil dry weight. Error bars indicate SE (n = 4). [file Image2.tif]

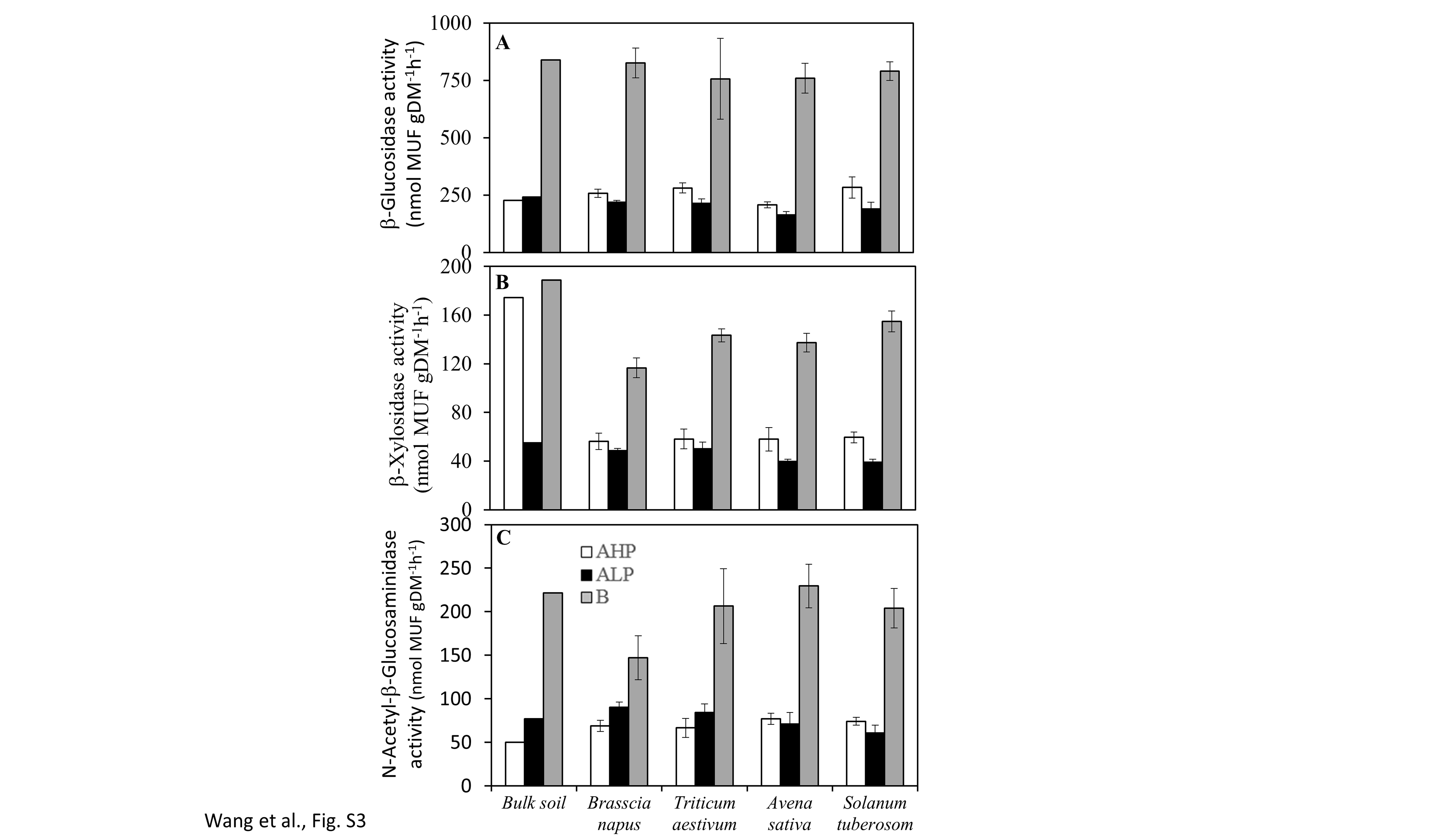

Supplement: Figure S3 — (A) β-glucosidase activity, (B) β-xylosidase activity, and (C) N-acetyl-β-glucosaminidase activity of bulk soil for Brassica napus, Triticum aestivum, Avena sativa, and Solanum tuberosum grown in soils AHP (white bars), ALP (black bars) and B (gray bars). Error bars indicate SE (n = 4). [file Image3.tif]

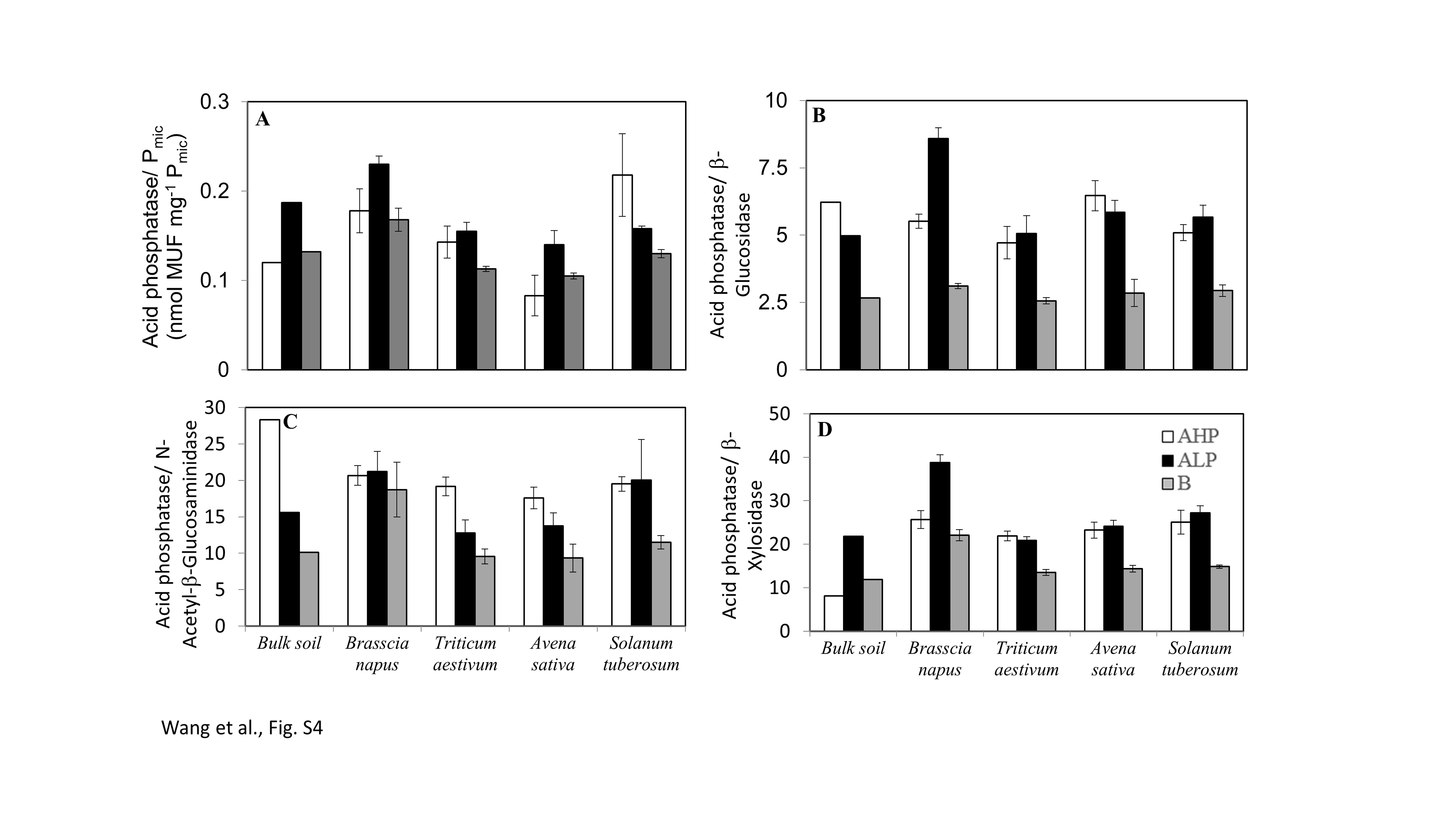

Supplement: Figure S4 — (A) Ratio of acid phosphatase activity to Pmic, (B) ratio of acid phosphatase activity to β-glucosidase activity, (C) ratio of acid phosphatase activity to N-acetyl-β-glucosaminidase activity, and (D) ratio of acid phosphatase activity to β-xylosidase activity of bulk soil, for Brassica napus, Triticum aestivum, Avena sativa, and Solanum tuberosum grown in soils AHP (white bars), ALP (black bars), and B (gray bars). Error bars indicate SE (n = 4). [file Image4.tif]
